# Sleep interruption aggravates sepsis by rewiring the macrophage immune response

**DOI:** 10.1093/jimmun/vkag130

**Published:** 2026-06-08

**Authors:** Jacob A Allen, Taniah Ali, Daniela Rodarte, Luiz Garcia, Mark E Murray, Mackenzie Morgan, Chidozie Ugochukwu, Anjali Patel, Sydney Ligon, Alok K Dwivedi, Wendy E Walker

**Affiliations:** Department of Biomedical Sciences, Mercer University School of Medicine, Columbus, GA, United States; Department of Molecular and Translational Medicine, Paul L. Foster School of Medicine, Texas Tech University Health Sciences Center El Paso, El Paso, TX, United States; Department of Molecular and Translational Medicine, Paul L. Foster School of Medicine, Texas Tech University Health Sciences Center El Paso, El Paso, TX, United States; Department of Molecular and Translational Medicine, Paul L. Foster School of Medicine, Texas Tech University Health Sciences Center El Paso, El Paso, TX, United States; Department of Biomedical Sciences, Mercer University School of Medicine, Columbus, GA, United States; Department of Biomedical Sciences, Mercer University School of Medicine, Columbus, GA, United States; Department of Biomedical Sciences, Mercer University School of Medicine, Columbus, GA, United States; Department of Biomedical Sciences, Mercer University School of Medicine, Columbus, GA, United States; Department of Molecular and Translational Medicine, Paul L. Foster School of Medicine, Texas Tech University Health Sciences Center El Paso, El Paso, TX, United States; Center for Integrated Biostatistics and Epidemiology, Department of Biomedical Informatics, Biostatistics, and Medical Epidemiology, Missouri School of Medicine, Columbia, MO, United States; Department of Biomedical Sciences, Mercer University School of Medicine, Columbus, GA, United States

**Keywords:** macrophage, mouse, sepsis, sleep, transcriptome

## Abstract

Sepsis is the leading cause of death in hospitals and is very common in intensive care units (ICUs). Sleep is frequently interrupted in the hospital setting, especially within the ICU. Patients who sleep poorly have worse outcomes, such as increased mortality and longer hospital stays; however, the molecular basis remains poorly understood. In this study, we utilized a mouse model to investigate the impact of sleep interruption on subsequent sepsis. We found that sleep interruption aggravated sepsis, as evidenced by higher mortality rates (88% in mice with interrupted sleep vs 57% in mice with normal sleep; *P* = 0.0045) and worse disease scores. This effect occurred in both females and males. Sleep interruption increased circulating T cells and CD8^+^ T-cell activation during sepsis. Sleep interruption also increased the levels of serum cytokines (including IL-23 before sepsis was induced, and IL-6, TNF-α, MCP-1, and IL-10 after sepsis), and amplified macrophage cytokine production ex vivo. These ex vivo effects were largely dependent on Toll-like receptor 2 (TLR2), and sleep interruption no longer exacerbated sepsis in TLR2 knockout mice. Interestingly, the effects of sleep interruption on sepsis were reversed by 48 hours of recovery sleep, consistent with a mechanism involving altered gene expression rather than epigenetic changes. RNA sequencing identified 680 genes that were significantly up- or downregulated in macrophages from animals subject to sleep interruption, including multiple genes related to pathogen defense and cytokine signaling. Our study confirms that good sleep is essential to maximize sepsis survival and provides insight into the molecular basis whereby poor sleep alters immune function.

## Graphical abstract

**Graphical Abstract:**
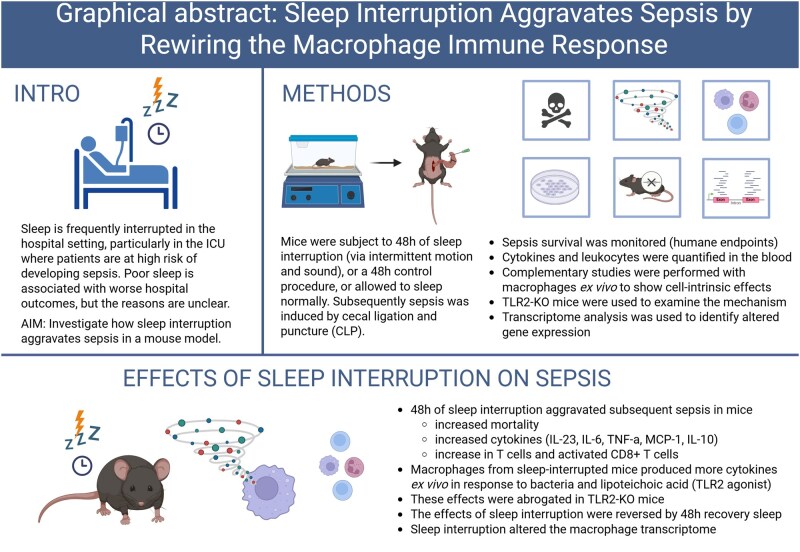

## Introduction

Sleep is a crucial restorative process for mental and physical health.^1^ Most animals sleep, although there are significant variations in sleep duration, timing, and architecture between species.^2^ The importance of sleep was demonstrated in rats, where total sleep deprivation was lethal to all animals within 2 to 3 weeks.^3^ On a fundamental level, sleep is defined by specific patterns of brain activity coupled with physical rest.^2^ Two processes intersect to induce sleep. First, the homeostatic drive for sleep increases with every waking hour (process S),^4^ which is accomplished in part through the buildup of adenosine.^5^ Second, circadian rhythms gate sleep propensity to the appropriate time of day, based on the light cycle and other environmental cues (process C),^4^ and accomplished in part by the altered sensitivity of adenosine receptors.^5^ The intersection of these 2 signals induces the sleeping state.^4^

This process, however, is imperfect since sleep is easily disturbed. In the hospital setting, and particularly within the intensive care unit (ICU), patients often experience interrupted sleep due to nighttime medical care, abnormal light patterns, and other factors, as highlighted in our review article.^6^ Poor sleep is associated with inflammatory diseases and altered immune function.^6^ Impaired sleep can also disrupt the normal circadian regulation of immune function over the course of the daily 24-hour cycle, due to the reciprocal feedback between sleep and the circadian clock.^6^

Sepsis is a condition that evolves when a severe infection induces dysregulated immunity.^7^ This leads to severe inflammation, immune suppression, and coagulopathy. Eventually, organ damage ensues, and the patient is at high risk of death. While sepsis can result from community-acquired infections, it is often nosocomial in its nature. Sepsis is a frequent complication of surgery and often evolves during an ICU stay in patients who have experienced burns or trauma. Our prior work showed that circadian rhythms influence sepsis, with mice developing worse disease when infection is induced in the daytime versus the nighttime, through a Toll-like receptor 2 (TLR2)–dependent mechanism.^8^^,^^9^ We reasoned that sleep may also play an important role in preserving optimal host defense against sepsis, leaving patients who sleep poorly at increased risk of death.

We undertook this study to determine if sleep interruption aggravates subsequent sepsis and to define the molecular mechanisms of this effect. We found that sleep interruption increased sepsis mortality in both female and male mice. Regarding the mechanism, sleep interruption increased macrophage cytokine production in response to sepsis-causing bacteria. These effects were largely dependent on TLR2. We further determined that sleep interruption induces substantial changes in the macrophage transcriptome, including genes related to the defense response to bacteria, cell killing, and cytokine–cytokine receptor interactions.

## Materials and methods

### Animals

All animal experiments were approved by the Institutional Animal Care and Use Committees at Mercer University School of Medicine and Texas Tech University Health Sciences Center (TTUHSC) El Paso. The study utilized C57BL/6, TLR2-KIGFP, and TLR2 knockout (KO) mice (all strains were C57BL/6 congenic). Breeders were obtained from The Jackson Laboratory (Bar Harbor, ME, USA) and progeny were produced in the vivariums at Mercer University School of Medicine and TTUHSC El Paso. Both male and female mice were included, and the animals were aged 5 to 16 weeks. Animal numbers for each experiment are described in the figure legends. Animals were housed in Techniplast emerald cages and fed Formulab Diet 5008 until 8 weeks of age, and then 5001 Rodent Diet thereafter (Both from LabDiet, St Louis, MO, USA). Animals were provided with water in bottles or via lixits ad libitum, except during the sleep interruption procedure as indicated below. The facilities maintained a 12-hour light/12-hour dark cycle.

### Sleep interruption

**Figure 1:**
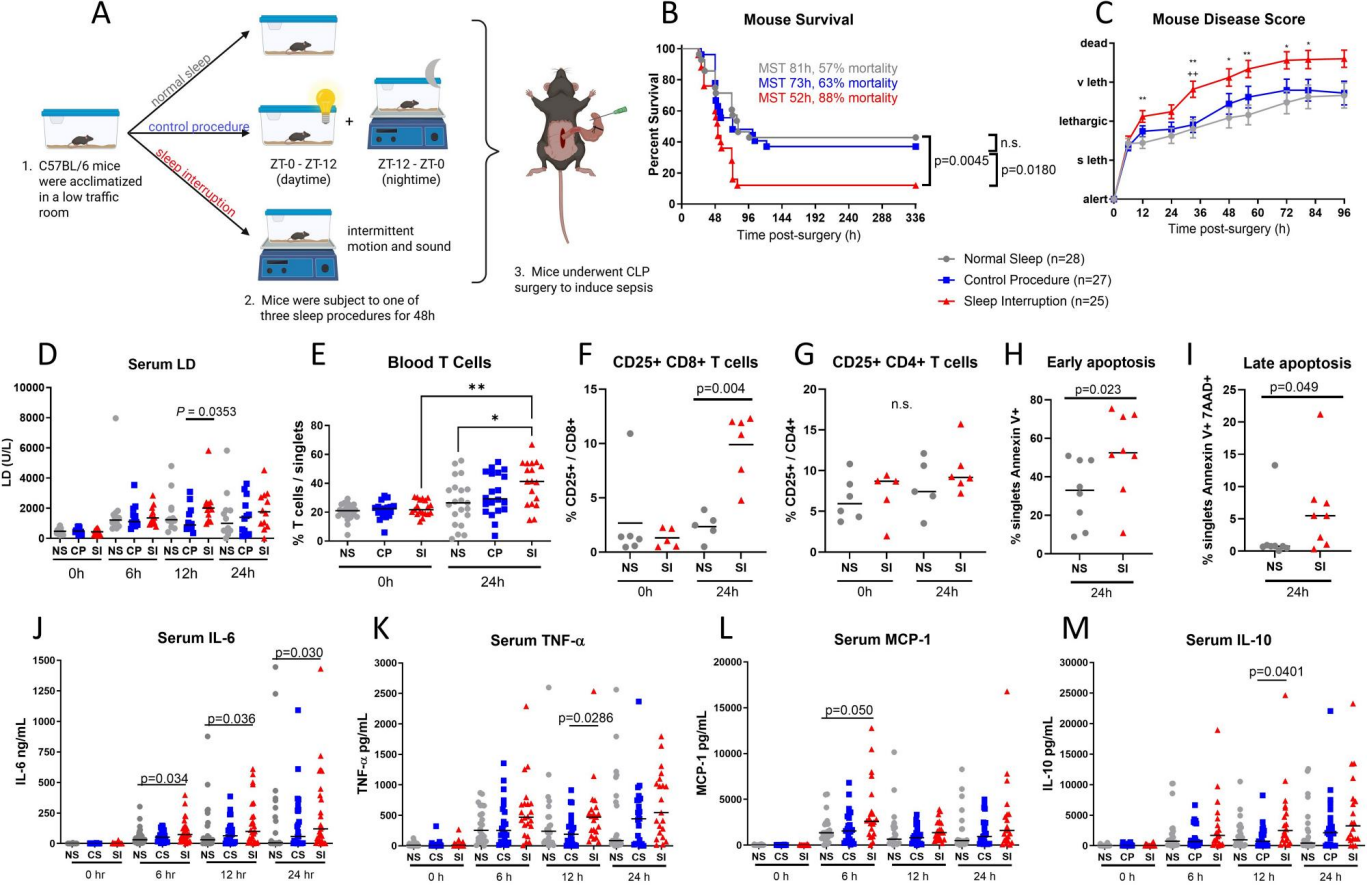
Sleep interruption aggravates sepsis in mice. (A) Mixed-sex C57BL/6 mice (*n* = 25–28/group) were subject to sleep interruption (SI), a control procedure (CP), or allowed normal sleep (NS), and subsequently sepsis was induced by CLP, per the scheme shown (*n* = 25–28/group). We monitored (B) mouse survival and (C) disease score, based on the degree of lethargy. Blood samples were obtained from the mice at 0 h (after SI, before CLP), and at 6, 12, and 24 h post-CLP. (D) We determined serum LD levels (via an enzyme kinetic assay). (E–G) We used antibody staining and FACS analysis (*n* = 6–8 animals/group) to determine (E) % CD3^+^ T cells among the leukocyte singlets, (F) % of CD8^+^ T cells that are CD25^+^, and (G) % of CD4^+^ T cells that are CD25^+^, at 0 h and 24 h post-CLP. Peritoneal lavage samples were obtained from animals subject to SI vs NS (*n* = 8/group), stained with Annexin V and 7-AAD, and then analyzed by FACS to quantify singlets that were (H) Annexin V single positive, representing early apoptosis, and (I) Annexin V, 7-AAD double positive, representing late apoptosis. Serum cytokines were quantified at each time point via a FACS-based multiplex bead assay (*n* = 25–32/group), including (J) IL-6, (K) TNF-α, (L) MCP-1, and (M) IL-10. Survival data were compared with log-rank tests, and *P* values are shown on the figure. Disease score was compared by 2-way ANOVA and Tukey multiple comparison test; *P < 0.05 for SI vs NS; **P < 0.01 for SI vs NS; ++ *P* < 0.01 for SI vs CP. Each data point represents a single biological replicate. Serum LD values, blood T-cell proportions, and cytokine data were compared with a Kruskal–Wallis test, followed by a Dunn multiple comparison test. Where the *P* value was <0.05, a Dunn multiple comparison test was also performed, and multiplicity-adjusted *P* values are shown on the figure with a bar to indicate the groups that differed. For pairwise comparison of T-cell activation and apoptosis (F–I), we used a Mann–Whitney test at each time point, and *P* values are shown on the figures. n.s., not significant.

We adapted a sleep interruption protocol described by Sinton et al., which was validated to reduce rapid eye movement (REM) and non-REM sleep in mice.^10^ The mouse cage was placed on an intermittent orbital rotator that was set to cycle on at 100 rpm for 30 seconds and then off for 90 seconds. A binder clip was attached to the cage to create an additional auditory tapping stimulus. Experimental mice were divided into 3 groups (Fig. 1A). Mice in the “sleep interruption” group were placed on this apparatus at zeitgeber time (ZT) 0 (eg 7:30 am; lights on) for 48 hours. Mice in the “normal sleep” group were housed in their normal stationary cages for 48 hours. Mice in the “control procedure” group were housed on the shaker during their active phase (dark period) and housed in stationary mode during their rest phase (light period) for 48 hours. All animals received hydrogels in place of their normal water bottles during this period. After these treatments (ZT-0), animals were subject to cecal ligation and puncture (CLP) or euthanized via cervical dislocation under isoflurane anesthesia, to obtain peritoneal macrophages (as described below). For the sleep recovery groups, animals were subject to the sleep interruption procedure described above and then housed in their normal stationary cages for the specified amount of time before inducing sepsis via CLP at ZT-0.

### Cecal ligation and puncture

We performed CLP to induce sepsis. The mice were first anesthetized with a precision isoflurane vaporizer. Appropriate anesthetic depth was verified and maintained throughout the procedure by monitoring the breathing and heart rate and confirming a lack of pedal reflex or any other responses to a toe pinch. Each animal was weighed and injected subcutaneously with buprenorphine SR (1 mg/kg; Wedgewood Pharmacy, Swedesboro, NJ, USA). The abdomen was shaved and scrubbed with povidone-iodine solution and then ethanol, 3 times. A petroleum-based lubricant was applied to protect the eyes. The animal was placed in the surgical area and covered with a square of press and seal surgical drape, with the abdomen exposed through a circular opening. An incision of ∼1 to 1.25 cm was made in the skin and underlying peritoneal membrane at the linea alba. The cecum was located with blunt forceps and brought to the exterior. Then, 1 cm of the distal portion was tied off with a black braided silk suture (Covetrus, Portland, ME, USA), and a 21-g needle was passed through the cecum (in and out, eg 2 punctures). The cecum was squeezed to extrude a small drop of its contents onto its exterior surface and then replaced into the abdominal cavity. The peritoneal musculature was closed with a running suture and the skin with an interrupted suture, both using purple Vicryl suture (Covetrus). Each animal was subcutaneously administered 1 mL of warm (37 °C) lactated Ringer’s solution postoperatively (Covetrus). Thermal support was provided via a heating pad during and after the surgery. Imipenem-cilastatin antibiotics (Covetrus) were administered intraperitoneally at a dose of 25 mg/kg, suspended in lactated Ringer’s solution (5 mL volume/kg body weight). This solution was administered twice daily starting several hours postoperatively, for a total of 9 doses. The first dose of antibiotics was administered 2 to 6 hours postoperatively, and this timing was adjusted to account for seasonal variations in model performance (described in our prior report^11^) in order to maintain a consistent survival rate throughout the study (25% to 50% in the normal sleep group).

### Blood collection and serum preparation

Blood was collected from the retro-orbital plexus or submandibular vein, as described in our prior reports^12^^,^^13^; at 0 hours (post–sleep interruption and prior to surgery); and at 6, 12, and 24 hours post-CLP. We found that submandibular bleeds obscured the accurate measurement of creatine kinase^13^; therefore, only retro-orbital blood samples were used for this analysis. To prepare serum, blood was placed in BD gold-top serum separator microtainers (Fisher Scientific, Hampton, NH, USA) and spun at 12,000 rpm 2”. Serum was collected from above the clot separator layer and stored at −80 °C. For whole blood staining, samples were placed in BD purple top K+ EDTA microtainers (Fisher Scientific) to prevent clotting.

### Peritoneal lavage, macrophage enrichment, and culture

Peritoneal lavage was performed with 3 mL PBS, as described previously.^12^ The cells were enumerated with a hemacytometer slide, and 0.5 × 10^6^ cells were seeded per well. The cells were then stimulated with a suspension of sonicated bacteria prepared from the mouse cecum, at a 1/20,000 dilution, or with 2 µg/mL lipoteichoic acid (LTA), LPS 1 µg/mL, or with media only (rest), as described in our prior reports.^8^^,^^14^^,^^15^ Four hours later, the cell culture media were collected and frozen at −80 °C.

### Antibody staining and FACS analysis of leukocyte populations

The methods for staining whole blood and peritoneal lavage cells and the FACS analysis methods are described in our prior reports.^16^^,^^17^ Fluorescence data were acquired with a FACS Canto II or Cytoflex S flow cytometer and analyzed with FlowJo software. We first gated cells based on their FSC:SSC profile, and then singlets based on their SSC-H versus SSC-A profile. We identified leukocytes by gating CD45^+^ events. Within this population, T cells (CD3^+^) and B cells (CD19^+^) were identified. For myeloid cells, CD11b^+^ events were gated within the leukocytes, and then monocytes (Ly6C^hi^, Ly6G^lo^), and neutrophils (Ly6C^hi^, Ly6G^hi^) were identified. To examine T-cell activation, we identified CD4^+^ and CD8^+^ T cells within the CD45^+^ gate and then analyzed CD25^+^ expression as a marker of their activation. For peritoneal lavage samples, we gated CD11b^+^ myeloid cells as described above, and then identified large peritoneal macrophages (LPMs; F4/80^hi^, MHCII^lo^) and small peritoneal macrophages (SPMs; F4/80^hi^, MHCII^lo^), per our prior report.^16^ We measured GFP expression within these subsets as a measure of the TLR2-KIGFP reporter gene. To measure apoptosis in peritoneal lavage cells, we used the FITC-Annexin V apoptosis detection kit with 7-aminoactinomycin D (7-AAD) (BioLegend, San Diego, CA, USA), per the manufacturer’s instructions.

### Measurements of organ damage

Creatine kinase (CK), lactate dehydrogenase (LD), and blood urea nitrogen (BUN) were measured in mouse serum samples using Pierce Scientific reagents (Fisher Scientific). The assay was adapted to measure these using a cuvette and a spectrophotometer.

### Cytokine measurements

We initially measured 13 cytokines in the C57BL/6 mouse sera using the mouse inflammation panel LegendPLEX bead-based assay (BioLegend). This assay was performed per the manufacturer’s instructions but utilizing half of the recommended volumes. We measured IL-6 in the mouse sera using BD OptEIA mouse IL-6 ELISA set (Becton Dickinson, Franklin Lakes, NJ, USA), due to its high levels. Further tests employed BD OPTEIA mouse IL-6, IL-23, MCP-1, TNF-α, and IL-10 ELISA sets (Becton Dickinson) and the Invitrogen mouse IL-23 ELISA set to measure cytokines in culture supernatants and TLR2-KO sera.

### Statistical analysis

Statistical analyses were performed with GraphPad software. Survival data were compared with a log-rank test and shown with a Kaplan–Meier curve. Disease score was compared with a 2-way analysis of variance (ANOVA) and a Tukey multiple comparison test. All other measures were compared with a Kruskal–Wallis test and Dunn multiple comparison test, where 3 or more groups were compared (eg sleep interruption, control procedure, and normal sleep), or a Mann–Whitney test where 2 groups were compared (eg sleep interruption vs normal sleep). Multiplicity-adjusted *P* values are shown on the graphs.

### RNA harvest, RNA sequencing, and transcriptome analysis

Adherent peritoneal macrophages were enriched from mice as described above. The cells were washed twice with PBS, and then 350 mL buffer RTL (QIAGEN, Germantown, MD, USA) was added to the dish, and the cells were scraped off the plate with a rubber policeman. RNA was subsequently prepared with an RNeasy mini kit (QIAGEN), according to the manufacturer’s instructions. The RNA yield and purity were confirmed with a spectrophotometer. Library construction, RNA sequencing (RNA-seq), and bioinformatic analyses were performed by Novogene (Sacramento, CA, USA). In brief, mRNA was purified using poly-T oligo-attached magnetic beads. First-strand cDNA synthesis was then completed using random hexamer primers, followed by second-strand synthesis using dTTP. This was followed by adapter ligation, size selection, amplification, and purification to create libraries. Libraries were sequenced on an Illumina NovaSeq X Plus instrument using nondirectional paired-end 150-bp reads, yielding an average of ∼6 Gb per sample. Reads from sequencing were trimmed, low-quality reads (Phred score <30) filtered out, and the remaining reads aligned to the *Mus musculus* reference genome (mm39) using HISAT2^18^ (v2.2.1). Gene-level counts were generated with featureCounts^19^ (v2.0.6) and normalized to fragments per kilobase per million mapped reads. Cluster analysis of normalized expression profiles was performed using k-means clustering based on Euclidean distance. Differential expression analysis was conducted in DESeq2^20^ (v1.42.0) using raw count data. *P* values were calculated using Wald statistics and adjusted for multiple testing with the Benjamini–Hochberg false discovery rate method. Genes with *P* value ≤0.05 and absolute log_2_ fold change ≥0 were considered differentially expressed. Differentially expressed genes (DEGs) were subsequently used for functional enrichment analysis in clusterProfiler^21^ (v4.8.1) to identify significantly enriched Gene Ontology (GO) terms (biological processes, molecular function, cellular components), as well as Reactome and Kyoto Encyclopedia of Genes and Genomes (KEGG) pathways. Enrichment was assessed using Fisher exact test with an adjusted *P* value cutoff of 0.05.

## Results

### Sleep interruption aggravates sepsis

We used a mouse model to determine if sleep interruption aggravates subsequent sepsis (Fig. 1A). Wild-type (WT) C57BL/6 mixed-sex mice were separated into 3 cohorts (*n* = 25–28/group). The first was subject to sleep interruption (via housing on an intermittent orbital rotator with sound stimulus), the second was subject to a control procedure (housed on the orbital rotator during their waking nighttime hours and returned to stationary housing during the daytime), and the third was allowed to sleep normally (never placed on the orbital rotator). Subsequently, abdominal sepsis was induced by CLP.

We found that animals with sleep interruption exhibited a significantly higher rate of mortality (88%) and a shorter median survival time (MST = 52 hours) in comparison to animals with normal sleep (57% mortality, MST = 81 hours; *P* = 0.0045), and relative to the control procedure group (63% mortality, MST = 73 hours; *P* = 0.0180) (Fig. 1B). To determine the effects of biological sex, we also stratified this group into females and males. We found that sleep interruption increased mortality in both female and male mice (Fig. S1A, B). We also measured the animals’ disease score (degree of lethargy) and found this to be significantly higher in animals subject to sleep interruption versus normal sleep (Fig. 1C). These data indicate that sleep interruption aggravates sepsis in mice.

The control procedure group was included in our study to identify any off-target effects of housing the mice on the intermittent orbital rotator. Notably, mice that underwent the control procedure before sepsis showed similar mortality and disease score to the normal sleep group (Fig. 1B). Moreover, we measured the levels of corticosterone (the major stress hormone in mice) following the sleep treatment and prior to the induction of sepsis. We observed similar levels of corticosterone in the normal sleep group (median, 93.39 [IQR, 16.41–132.7]), the control procedure (median, 74.31 [IQR, 28.56–119.8]), and the sleep interruption procedure (median, 121.9 [IQR, 84.91–181.7]), with no significant differences between the groups (Fig. S2). These data indicate that the orbital housing procedure did not induce significant levels of stress in the mice and ruled out off-target effects of the housing procedure.

### Sleep interruption worsens tissue damage during sepsis

Organ dysfunction is a defining feature of sepsis.^7^ Blood samples were obtained after the sleep interruption procedure, prior to CLP surgery (0 hours), and again at 6, 12, and 24 hours after CLP (or the equivalent timepoint in controls). We measured LD, indicative of tissue damage; BUN, indicative of kidney damage; and CK, indicative of muscle damage. In all 3 groups, LD (Fig. 1D), BUN (Fig. S3A), and CK (Fig. S3B) became elevated at 6 to 12 hours after sepsis was induced. In comparison to the mice that slept normally, animals subject to sleep interruption exhibited significantly higher levels of LD at 12 hours post-CLP (Fig. 1D), indicating a greater degree of tissue damage, as well as a trend toward higher levels of LD (Fig. 1D), BUN (Fig. S3A), and CK (Fig. S3B) at 24 hours post-CLP. These data indicate that sleep interruption exacerbates tissue damage during sepsis.

### Sleep interruption increases the frequency of circulating T cells and CD8^+^ T-cell activation

Bloodborne leukocytes are a critical component of host defense, and their numbers undergo significant shifts during sepsis due to emergency myelopoiesis,^22^ trafficking to sites of infection or inflammation,^23^ and apoptosis.^24^^,^^25^ To investigate how sleep affects this process, blood samples were obtained from animals subject to sleep interruption, the control procedure, or normal sleep at 0 and 24 hours post-CLP. Leukocyte populations were quantified in the bloodstream, including T cells, B cells, monocytes, and neutrophils. For all 3 groups, the percentage of T cells in the blood became more variable at 24 hours post-CLP in comparison to the presurgical levels, and the sleep interruption group showed a significant increase in T cells (Fig.1E). Notably, at 24 hours post-CLP, the percentage of blood T cells was significantly higher in the group subject to sleep interruption versus the normal sleep group (Fig. 1E). The ratio of CD4/CD8 T cells was not significantly altered by sleep or sepsis (Fig. S3C). We also examined CD25 (IL-2 receptor α), as a marker of T-cell activation and observed a significant increase in CD25^+^CD8^+^ T cells in the sleep interruption group versus the normal sleep group at 24 hours post-CLP (Fig. 1F). In contrast, sleep interruption did not substantially affect the percentage of CD25^+^CD4^+^ T cells (Fig. 1G).

We also quantified B cells, monocytes, and neutrophils in the bloodstream of these mice (Fig. S3D–F). For all 3 groups, the percentage of blood B cells showed a significant decrease at 24 hours post-CLP in comparison to presurgical levels (Fig. S3D), while blood monocytes became more variable and significantly increased (Fig. S3E). In all 3 groups, the percentage of neutrophils became more variable at 24 hours post-CLP in comparison to the presurgical levels and were significantly increased in the control procedure and sleep interruption groups at 24 hours in comparison to the presurgical levels, with a similar trend in the normal sleep group (Fig. S3). However, sleep interruption did not significantly affect the percentage of B cells, monocytes, or neutrophils preoperatively, nor at 24 hours postsepsis. In summary, sleep interruption alters the composition of blood leukocytes during a subsequent sepsis challenge, increasing the percentage and activation of CD8^+^ T cells.

### Sleep interruption increases leukocyte apoptosis during sepsis

Apoptosis of immune cells is a key factor in the pathophysiology of sepsis. To test how sleep interruption influences sepsis-induced apoptosis, animals were subjected to sleep interruption versus normal sleep for 48 hours, followed by CLP to induce sepsis. Twenty-four hours after surgery, the animals were euthanized, and peritoneal lavage was obtained. The peritoneal leukocytes were stained with Annexin V and 7-AAD to identify apoptotic cells via FACS analysis. We found that sleep interruption increased the proportion of peritoneal leukocytes in both early apoptosis (Annexin V single positive; Fig. 1H) and late apoptosis (Annexin V, 7-AAD double positive; Fig. 1I), in comparison with animals that slept normally.

### Sleep interruption increases systemic cytokine production during sepsis

During sepsis, a “cytokine storm” ensues. These secreted mediators exert pleiotropic effects that can be helpful or detrimental to the host, depending on the timing and levels.^26^ We obtained serum samples at 0, 6, 12, and 24 h post-CLP and measured the levels of 12 cytokines and chemokines. IL-23 showed a small but significant elevation in animals subject to sleep interruption versus the control procedure, prior to surgery (Fig. S4A). We observed that IL-6 (Fig. 1J), TNF-α (Fig. 1K), MCP-1 (Fig. 1L), and IL-10 (Fig. 1M) became elevated during sepsis, with significantly higher levels in animals that were subject to sleep interruption versus normal sleep, and versus the control procedure (*P* values are noted in the figures, with a bar to indicate the groups that differed in the multiple comparison tests). IL-23, IL-17A, IL-1β, IL-12p70, IFN-β, IFN-γ, IL-27, and GM-CSF were also elevated in septic animals (Fig. S4A–H), and we observed some trends toward higher cytokine levels in the sleep interruption group versus the control groups. In summary, sleep interruption broadly increased the systemic cytokine response to sepsis, affecting both proinflammatory and immunosuppressive cytokines.

### Sleep interruption increases macrophage cell-intrinsic cytokine response, dependent on TLR2

**Figure 2:**
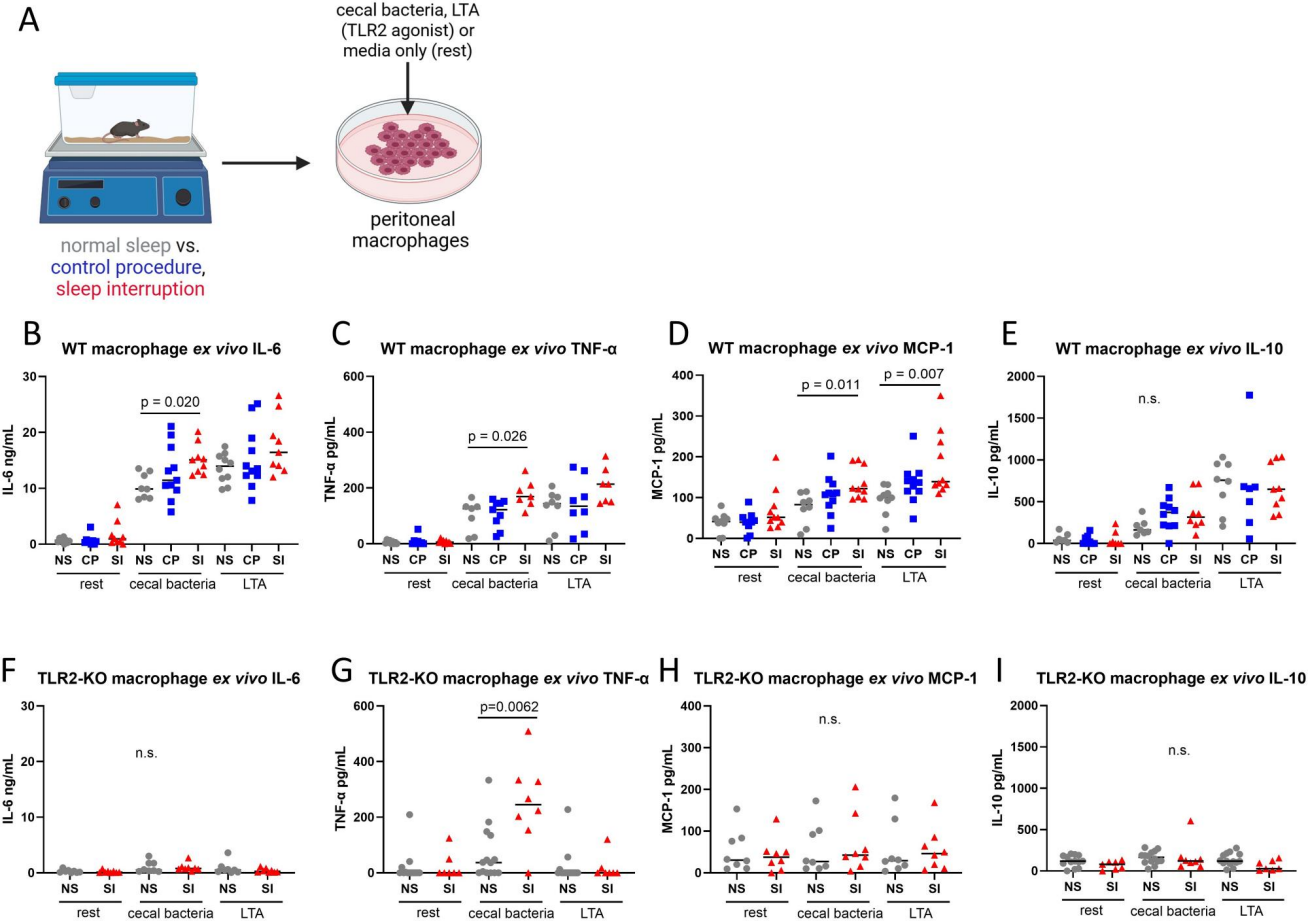
Sleep interruption increases macrophage production of cytokines ex vivo, with TLR2-dependent effects. (A) Male and female C57BL/6 mice (*n* = 7–10/group) were subject to sleep interruption (SI), a control procedure (CP), or allowed normal sleep (NS). Peritoneal macrophages were harvested and cultured ex vivo with sonicated cecal bacteria, LTA, or media only (rest) for 4 h. The media was harvested after 4 h and we used ELISA to quantify (B) IL-6, (C) TNF-α, (D) MCP-1, and (E) IL-10. The experiment was repeated with TLR2-KO mice (SI and NS; *n* = 8/group), and we quantified (F) IL-6, (G) TNF-α, (H) MCP-1, and (I) IL-10. Each data point represents a single biological replicate. For (B–E) (3 groups), data were compared with a Kruskal–Wallis test at each time point. Where the *P* value was <0.05, a Dunn multiple comparison test was also performed, and multiplicity-adjusted *P* values are shown on the figure with a bar to indicate the groups that differed. For pairwise comparison of TLR2-KO mice (F–I), we used a Mann–Whitney test at each time point, and *P* values are shown on the figures. n.s., not significant.

Peritoneal resident macrophages produce cytokines in response to bacteria^8^^,^^14^ and play a key role in host defense during sepsis.^16^ We reasoned that sleep interruption may directly increase the capacity of macrophages to produce cytokines. To test this, C57BL/6 mice were subject to sleep interruption, the control procedure, or normal sleep. Subsequently, the animals were euthanized, and peritoneal lavage was harvested. The macrophages were enriched and cultured in the presence of sonicated bacteria derived from the cecum, LTA (a TLR2 agonist), or media only (rest) for 4 hours before quantifying cytokines in the media (Fig. 2A). Macrophages from animals subject to sleep interruption produced significantly more IL-6 (Fig. 2B), TNF-α (Fig. 2C), and MCP-1 (Fig. 2D) in response to cecal bacteria, in comparison to macrophages harvested from animals that slept normally. A similar trend was noted for IL-10 (Fig. 2E). A similar finding was obtained when the macrophages were stimulated with LTA, which induced significantly more MCP-1 (Fig. 2D), and a trend toward more IL-6 (Fig. 2B) and TNF-α (Fig. 2C) in mice subject to sleep interruption versus normal sleep, providing a clue about the mechanism. These changes were not replicated in animals subject to the control procedure, suggesting a sleep-specific effect. Also, cultured macrophages from animals subject to sleep interruption produced significantly more IL-6 in response to LPS (TLR4 agonist), suggesting that sleep interruption modulates multiple TLR pathways (Fig. S5). Notably, in this repeat set of experiments, sleep interruption significantly increased the IL-6 response to LTA (TLR2 response; Fig. S5).

To determine if TLR2 mediated the cytokine response to cecal bacteria, we repeated this experiment using TLR2-KO mice that were subject to sleep interruption or allowed to sleep normally. We found that TLR2-KO macrophages produced little IL-6 (Fig. 2F), MCP-1 (Fig. 2H), or IL-10 (Fig. 2I) in response to cecal bacteria or LTA. In contrast, TNF-α was still produced in response to cecal bacteria, and the levels were elevated in macrophages derived from animals subject to sleep interruption versus normal sleep (Fig. 2G). Our data reveal that macrophage cytokine production in response to cecal bacteria is largely TLR2-dependent, apart from TNF-α.

### The effects of sleep interruption on sepsis are TLR2 dependent

**Figure 3:**
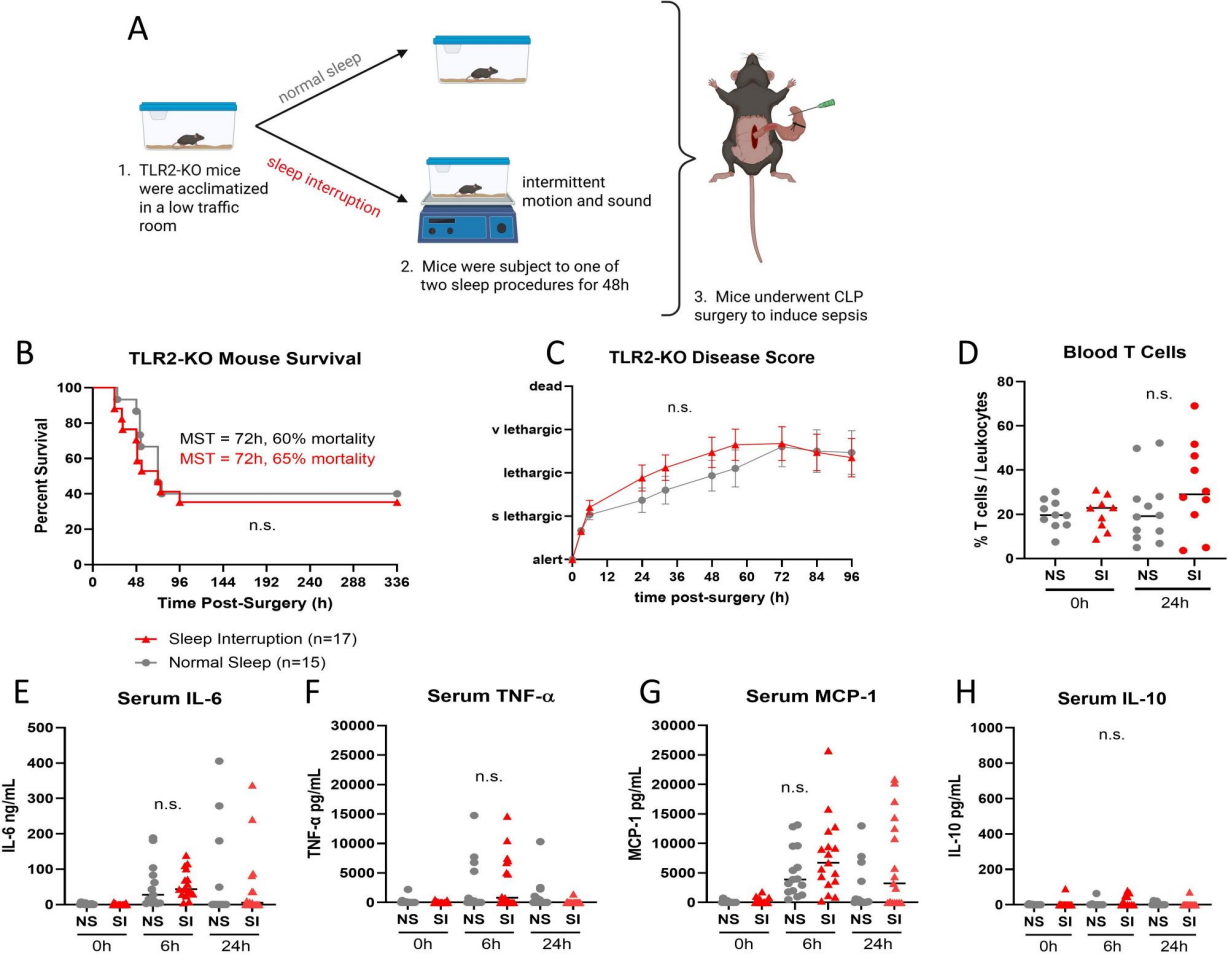
The effects of sleep interruption on sepsis are abrogated in TLR2-deficient mice. (A) Mixed-sex TLR2-KO mice (*n* = 15–17/group) were subject to sleep interruption (SI) or allowed normal sleep (NS), and subsequently sepsis was induced by CLP. We monitored (B) mouse survival and (C) disease score, based on the degree of lethargy. (D) The percentage of CD3^+^ T cells was determined in the blood via antibody staining and FACS. Serum cytokines were quantified via ELISA at 0 h (after SI, before CLP), and at 6, 12, and 24 h post-CLP, including (E) IL-6, (F) TNF-α, (G) MCP-1, and (H) IL-10. Each data point represents a single biological replicate. Survival data were compared with log-rank tests, disease score was compared by 2-way ANOVA, and other measures were compared with a Kruskal–Wallis test and Dunn multiple comparison test (no significant differences were observed). n.s., not significant.

We subsequently examined whether TLR2 is required for sleep interruption to exacerbate sepsis. TLR2-KO mice were subject to sleep interruption or normal sleep, and then sepsis was induced via CLP (Fig. 3A). In contrast to our results in WT mice, we found that TLR2-KO mice exhibited a similar sepsis phenotype, regardless of whether they had experienced sleep interruption or normal sleep (Fig. 3). The animals subject to sleep interruption exhibited a similar incidence and tempo of mortality (MST = 72 hours, 65% mortality), in comparison to animals that slept normally (MST = 72 hours, 60% mortality) (Fig. 3B). Although there was a slight trend toward higher disease scores at 24 to 60 hours, this difference was not significant and resolved by 72 hours (Fig. 3C). Unlike their WT counterparts, the TLR2-KO mice subject to sleep interruption did not show a significant elevation in blood T cells at 24 hours post-CLP in comparison to TLR2-KO mice that slept normally (Fig. 3D). In TLR2-KO mice, IL-6 (Fig. 3E), TNF-α (Fig. 3F), and MCP-1 (Fig. 3G) were still produced in response to sepsis, but there were no significant differences between the sleep interruption and normal sleep mice. Furthermore, little IL-10 was produced in septic TLR2-KO mice (Fig. 3H). These data indicate that TLR2 is required for sleep interruption to exert its full effects on sepsis outcomes.

We reasoned that sleep interruption may directly upregulate TLR2 on peritoneal macrophages. To test this, we used the TLR2KI-GFP strain, which contains an IRES-GFP sequence at the end of the TLR2 gene.^27^ These animals were subjected to sleep interruption for 48 hours or allowed to sleep normally, and then their peritoneal cells were harvested (Fig. S6A). As described in our prior publication,^16^ there are 2 populations of macrophages that reside in the peritoneal cavity: LPMs (CD45^+^, CD11b^+^, F48/80^hi^, MHCII^lo^) and SPMs (CD45^+^, CD11b^+^, F4/80^lo^, MHCII^hi^). We stained the cells with antibodies to identify these cells and then measured the GFP signal, via FACS. We found that TLR2 expression was not elevated in the LPM (Fig. S6B) or SPM (Fig. S6C) populations of animals subject to sleep interruption versus those that slept normally. Therefore, the increased cytokine production is not accomplished via upregulation of TLR2 itself, but via a different mechanism that influences this pathway.

### The effects of sleep interruption on sepsis are short-lived

**Figure 4:**
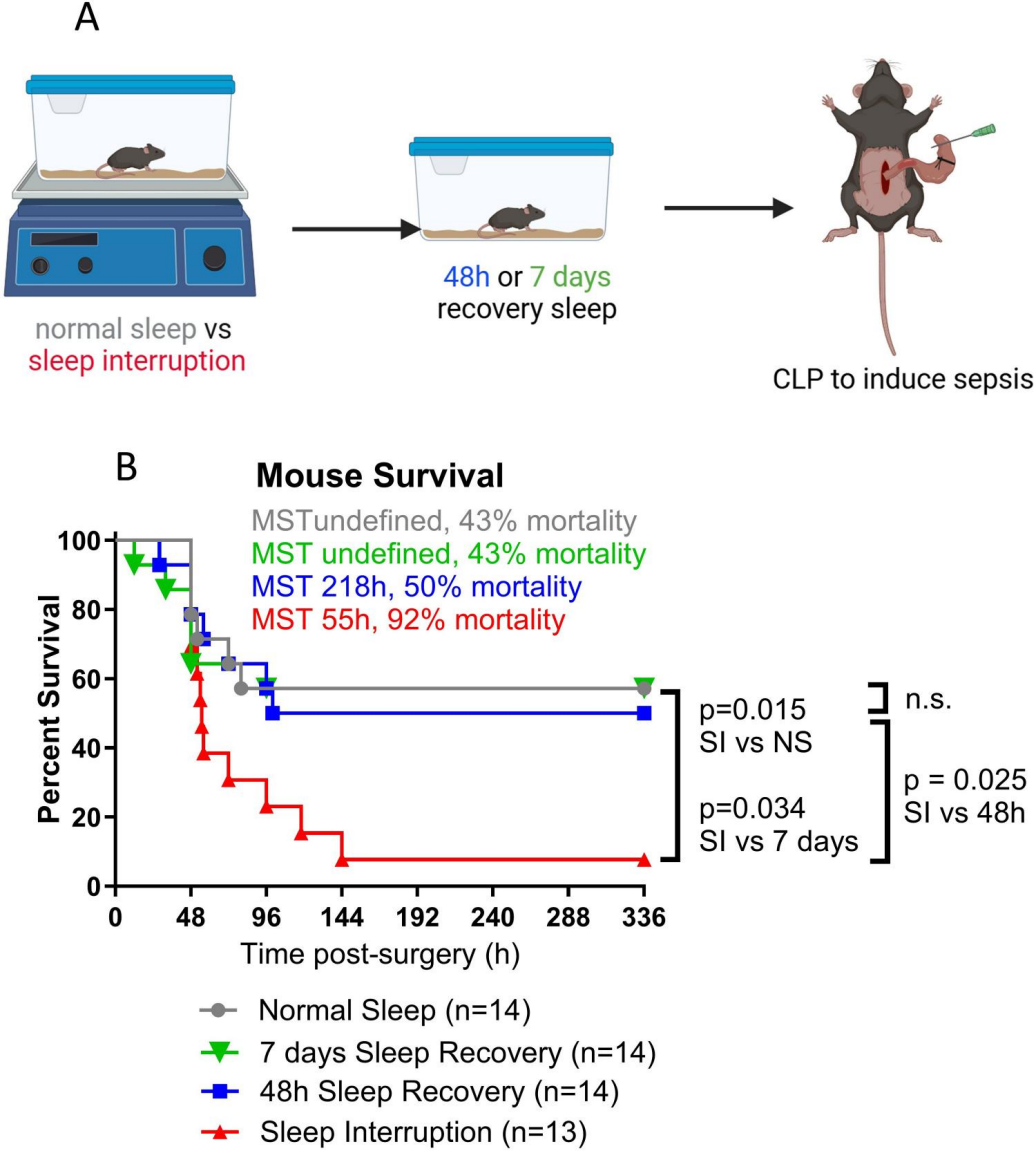
The effects of sleep interruption on sepsis are rapidly reversed by recovery sleep. (A) Male and female C57BL/6 mice (*n* = 13–14/group) were subject to sleep interruption (SI) and then allowed 48 h or 7 d of recovery sleep, prior to CLP. Additional control groups were subject to CLP immediately after sleep interruption or after normal sleep (NS). (B) We monitored animal survival. Data were compared with log-rank tests, and *P* values are shown on the figure. n.s., not significant.

Sleep interruption could potentially affect sepsis through simple changes in gene expression, which are quickly reversed, or via epigenetic changes, which last longer. To distinguish between these scenarios, we tested the duration of the effect. Mice were subject to sleep interruption and then allowed “recovery sleep” for 48 hours or 7 days before sepsis was induced. A control group was subject to sleep interruption immediately before sepsis, and another control group was allowed to sleep normally (Fig. 4A). We found that 48 hours of recovery sleep almost completely reversed the effects of sleep interruption on sepsis, as this group had a 50% mortality rate and that was similar to animals with normal sleep (43% mortality) (Fig. 4B). Furthermore, 7 days of recovery sleep fully reversed the effects of sleep interruption (43% mortality) (Fig. 4B). Notably, a log-rank test showed no significant differences in animal survival between these 3 groups. In contrast, animals subject to sleep interruption without recovery sleep had a much higher mortality rate (92%) and were significantly different from each of the other 3 groups (Fig. 4B; *P* values are shown in the figure). These data demonstrate that the effects of sleep interruption on sepsis are largely short-lived. Our result is consistent with the notion that sleep interruption predominantly influences sepsis through simple changes in gene expression, rather than epigenetic changes.

### Sleep interruption rewires the macrophage transcriptome

**Figure 5:**
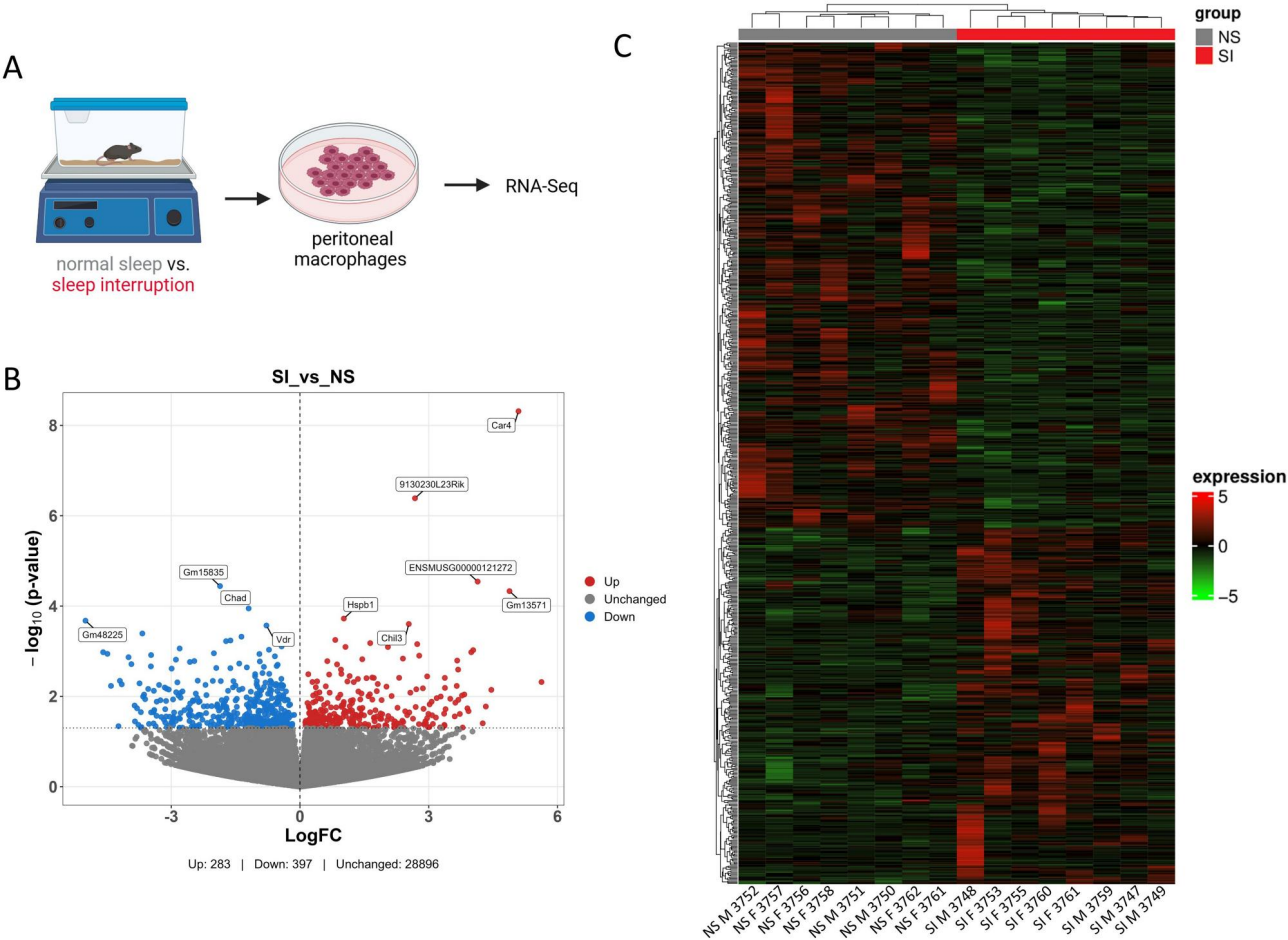
Sleep interruption rewires the macrophage transcriptome. (A) C57BL/6 mice (*n* = 8/group) were subject to sleep interruption (SI) or allowed normal sleep (NS). Subsequently, RNA was obtained from peritoneal macrophages, and bulk RNA-seq analysis was performed. (B) Volcano plot showing DEGs in SI vs NS mice, and the top 10 DEGs are labeled with the gene symbol. (C) Differential gene expression clustering heatmap.

To more broadly explore how sleep interruption changes macrophage gene expression, we performed an unbiased transcriptome analysis using RNA-seq. C57BL/6 mice were subject to sleep interruption for 48 hours or allowed to sleep normally. Immediately afterward, we collected peritoneal lavage and enriched the macrophages. Total RNA was harvested and an RNA-seq analysis was performed (Fig. 5A). We found that 680 genes showed differential expression between the groups, including 397 genes that were downregulated following sleep interruption and 283 genes that were upregulated following sleep interruption; a volcano plot is shown to illustrate the fold change and statistical significance (Fig. 5B). The top 10 DEGs highlighted in sleep interrupted mice versus normal sleep mice are labeled on the plot and the most highly upregulated/significant gene was carbonic anhydrase 4 (Car4). A cluster analysis was also performed to identify genes and gene sets with similar expression patterns (Fig. 5C).

**Figure 6:**
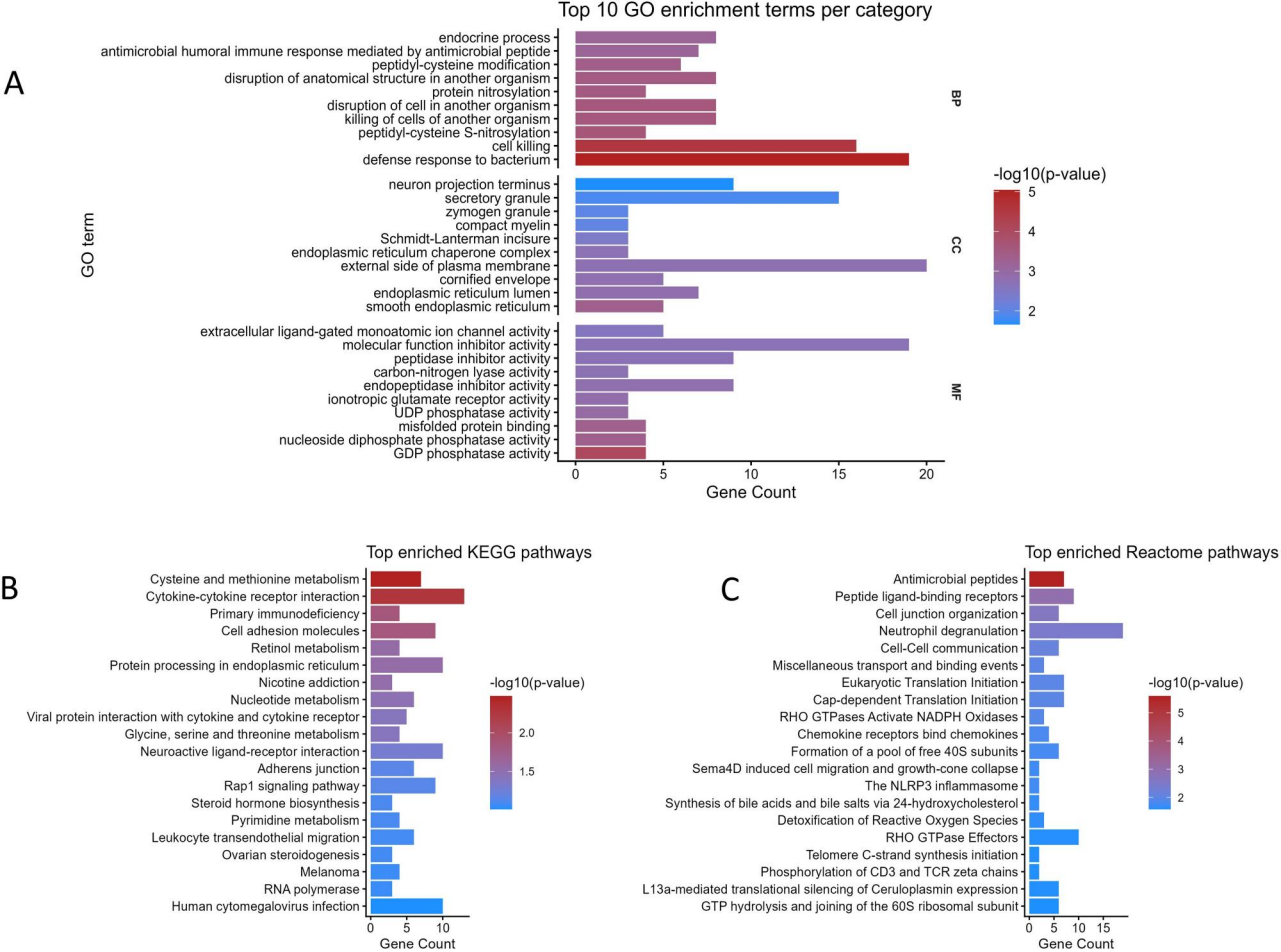
Enrichment analysis of DEGs reveals common pathways affected by sleep interruption. C57BL/6 mice (*n* = 8/group) were subject to sleep interruption or allowed normal sleep. Subsequently, RNA was obtained from peritoneal macrophages, and bulk RNA-seq analysis was performed (DEGs are shown in Figure 5). Enrichment analysis was performed on the DEGs to gain a deeper understanding of how they influence macrophage biology. (A) GO enrichment analysis barplot, with genes relating to biological processes (BP), cellular components (CC), and molecular function (MF), and only the first 10 GO terms for each category shown. (B) KEGG enrichment analysis barplot showing the first 20 pathways. (C) Reactome enrichment analysis barplot showing first 20 pathways. In all instances, the terms and pathways were ranked by adjusted *P* values, with a cutoff of ≤0.05.

GO enrichment analysis was employed to identify genes relating to specific biological processes, cellular components, and molecular function. The most significant 30 terms were selected for display (Fig. 6A). Of particular interest, we noticed altered gene expression relating to the defense response to bacterium (19 genes) and cell killing (16 genes); these genes and their functions are listed in Tables S1 and S2, respectively, alongside the fold change in expression and *P* values.

KEGG enrichment analysis identified genes involved in specific biological functions (Fig. 6B). This revealed differential regulation of genes relating to cysteine and methionine metabolism (7 genes) and cytokine–cytokine receptor interactions (13 genes). Tables S3 and S4 show a list of these genes, a description of their functions, the fold change in expression, and *P* values.

Reactome pathway enrichment revealed genes related to antimicrobial peptides (7 genes; Fig. 6C). The specific genes, fold change in expression, and *P* values are shown in Table S5. In summary, our data reveal that sleep interruption induces significant changes in gene expression relating to multiple pathways and biological processes in macrophages. These appear to underlie the increased cytokine production and heightened risk of sepsis death that occurs in mice with interrupted sleep.

## Discussion

It is well known that sleep affects many components of human physiology and is crucial for good health.^1^ In the hospital, and particularly the ICU, medical care is needed around the clock to ensure patients’ well-being. Paradoxically, this care repeatedly interrupts their sleep, with the potential to elicit harmful effects.^6^ Patients who sleep poorly in the hospital tend to have worse outcomes including increased risk of delirium,^28–30^ prolonged ventilation,^31^^,^^32^ greater severity of encephalopathy,^33^ longer hospital stays,^32^ increased hospital mortality,^33^ and functional impairments after discharge.^34^ However, it can be challenging to establish cause and effect in this context, because sleep changes are also induced by bacterial components^35^ and inflammatory mediators,^36^ defined as “sickness behavior.”^37^ Therefore, an important knowledge gap remains regarding how external sleep interruption affects critical illnesses. Our study was designed to address this topic and specifically examine how sleep affects sepsis, which is a leading cause of death in hospitalized patients, particularly for those who receive ICU care.^38^ Here, we provide direct evidence that repetitive sleep interruption induces significant alterations in the immune response, culminating in increased death in an animal model of sepsis. Animals whose sleep was interrupted before CLP showed higher mortality (Fig. 1B), paired with greater lethargy (Fig. 1C) and tissue damage (Fig. 1D). Notably, one prior report by Friese et al. obtained similar findings.^39^ In that manuscript, young (2 month) and middle-aged (9 month) male mice were subject to sleep interruption after CLP. The authors found that sleep interruption following septic challenge increased animal mortality.^39^ Using a slightly different approach (sleep interruption before septic challenge), we obtained similar findings. We demonstrated that this effect extends to both females and males (Fig. S1A, B) and expanded on this finding to explore the mechanism whereby sleep alters sepsis.

Was this finding an artifact of stress, or other off-target effects from being housed on the intermittent orbital rotator? To address this, our study included a control procedure group, where the animals were housed in the same manner as the sleep interruption mice, but only during the nighttime (when mice are typically awake) and returned to stationary housing during the daytime (when mice typically sleep). The control procedure group showed a similar sepsis phenotype to the normal sleep group (Figs. 1 and 2). These data suggest that the effects we observed in the sleep interruption group relate to the reduced sleep they experienced and are not off-target effects. Also, the serum corticosterone levels were similar in mice subject to normal sleep, sleep interruption, or the control procedure (Fig. S2), and low in all groups in comparison to levels induced by 24 hours of restraint stress in a prior report (∼1,000 ng/mL).^40^ These data indicate that the intermittent orbital rotator procedure used in our study did not induce a significant degree of stress in the mice.

How does sleep interruption affect the composition of blood leukocytes, both at baseline and in response to sepsis? Prior studies have found that sleep and circadian rhythms have a significant impact on circulating leukocytes, but these patterns are disrupted in the context of suboptimal sleep (discussed in more detail in our review article^6^). Circulating naïve T cells show a peak in the nighttime and a nadir in the daytime, in both rodents and humans.^41–43^ Furthermore, sleep disruption increased the peak numbers of CD4^+^ and CD8^+^ T cells.^42^^,^^44^^,^^45^ Additionally, T cells harvested at night exhibited greater proliferative capacity, and sleep deprivation further enhanced this response.^44^ In our study, sleep interruption alone did not significantly alter the composition of the blood leukocytes in the absence of sepsis. During sepsis, the populations of leukocytes in the bloodstream shift when a severe infection elicits emergency myelopoiesis, increasing stem cell turnover and favoring the generation of myeloid cells and their immature myeloid-derived suppressor cell counterparts.^22^ Sepsis also induces leukocyte migration to sites of infection and inflammation^23^ as well as widespread leukocyte apoptosis, compromising immune function.^24^^,^^25^

We observed that the percentage of B cells in the blood dropped significantly after sepsis was induced, while the percentage of monocytes and neutrophils increased and became more variable after sepsis induction (Fig. S3D–F). This effect was observed within every group (normal sleep, sleep interruption, and control procedure), and all were significant except for neutrophils in the normal sleep group, which still showed a strong trend. Notably, sleep interruption did not significantly change the percentage of B cells, monocytes, or neutrophils in the blood preoperatively, nor at 24 hours after CLP (Fig. S3D–F). Therefore sepsis, rather than sleep interruption, had a dominant effect on B-cell, monocyte, and neutrophil numbers in our study.

The percentage of T cells in the blood became much more variable after sepsis was induced (Fig. 1E). We observed that sleep interruption significantly increased the percentage of T cells at 24 hours postsepsis, both in comparison to their preoperative values and in comparison to the normal sleep group at 24 hours postsepsis (Fig. 1E). Therefore, sleep interruption and sepsis induced synergistic effects increasing T cells. Further analysis revealed that sleep interruption substantially increased the percentage of activated CD8^+^ T cells 24 hours after the infection was initiated (Fig. 1F). Our data are consistent with the literature describing increased numbers and activation of T cells with poor sleep^42^^,^^44^^,^^45^; however, for our study this effect was only revealed once infection occurred. This may be because our study used a milder sleep interruption protocol, in comparison to the more extreme sleep deprivation protocols used in those prior reports.^42^^,^^44^^,^^45^

How does sleep interruption affect leukocyte apoptosis? Prior studies showed that sepsis induces apoptosis of lymphocytes, including B cells and T cells.^24^^,^^25^ Furthermore, in patients with septic shock, greater apoptosis is associated with worse outcomes.^46^ We found that sleep interruption increased the apoptosis of peritoneal immune cells (Fig. 1H, I). In the future, it will be important to characterize the mechanism, for example, by examining how sleep interruption influences the expression of programmed death-1 (PD1) and its ligand programmed death ligand-1 (PD-L1), which are key drivers of sepsis-induced apoptosis.^47^^,^^48^

How does sleep interruption affect cytokine levels, at baseline and in response to sepsis? Prior research showed that the levels of serum IL-6 and its receptor (IL-6R) oscillate with the daily cycle in healthy human volunteers, albeit remaining at fairly low levels.^6^^,^^49^^,^^50^ Sleep deprivation altered this pattern and increased IL-6 and IL-6R as well as TNF-α.^6^^,^^49^^,^^50^ We performed a multiplex bead-based assay, complemented by ELISA, to broadly explore how cytokine levels were altered by sleep interruption both before and after sepsis. Prior to the induction of sepsis, sleep interruption increased the levels of serum IL-23 (Fig. S4A). This cytokine is a key mediator of inflammation, in large part through the induction of IL-17.^51^ We also observed a trend toward higher IL-17 at 24 hours postsurgery in animals subject to sleep interruption (Fig. S4B). These data are consistent with the notion that sleep interruption could potentially promote a Th17 response through the action of IL-23, in conjunction with IL-6 and TGF-β. In support of this notion, Liu et al. observed that sleep loss promoted Th17 differentiation in human and mouse cells and exacerbated an animal model of experimental autoimmune uveitis.^52^ IL-23 also promotes neutrophil recruitment and the production of inflammatory cytokines and chemokines, independent of IL-17,^53^ providing another potential mechanism whereby this cytokine may exacerbate sepsis. Notably, IL-23 blockade protected aged mice against sepsis.^54^

Sleep interruption also induced significant changes in the cytokine response to sepsis, increasing production of IL-6, TNF-α, and MCP-1 (Fig. 1J–L). IL-6 is a classic inflammatory cytokine that stimulates fever and the acute phase response and enhances T- and B-cell responses.^55^ While this cytokine is essential for host defense, excessive levels of IL-6 result in greater mortality rates during sepsis.^56–59^ TNF-α is a cardinal inflammatory cytokine that induces inflammatory gene expression and cell death^60^ and plays a critical role in the pathogenesis of inflammatory diseases.^61^ MCP-1 is a chemoattractant that recruits inflammatory monocytes to the site of infection,^61^ which amplify inflammation to detrimental levels in severe sepsis.

Conversely, sleep interruption also upregulated IL-10 after CLP (Fig. 1M). This cytokine dampens inflammation and promotes immunoparalysis. Notably, high IL-10 levels correlate with increased mortality in patients with sepsis,^26^^,^^62–66^ and abrogating IL-10 improves sepsis outcomes in mice.^67^ This is likely because of its action suppressing the protective functions of leukocytes. In summary, sleep interruption altered the cytokine milieu in a manner that is expected to induce harmful effects, promoting both inflammation and immunosuppression.

Does sleep interruption directly influence macrophage cytokine production, or is this a downstream effect when sepsis is made worse? Using an ex vivo culture system, we showed that sleep interruption directly increases the capacity of macrophages to produce IL-6, MCP-1, and TNF-α in response to bacteria (Fig. 2B–D); to produce IL-6 and MCP-1 in response to LTA (Fig. 2B, C and Fig. S5); and to produce IL-6 in response to LPS (Fig. S5). These data imply that sleep interruption affects cytokine production through macrophage-intrinsic effects, influencing multiple TLR pathways.

Deficiency of TLR2 greatly reduced the capacity of the macrophages to produce IL-6, MCP-1, and IL-10 in response to the bacteria and abrogated the amplifying effects of sleep interruption on cytokine production (Fig. 2F, H, I). Surprisingly, TLR2-KO macrophages still produced TNF-α in response to bacteria, and this was amplified by sleep interruption, akin to the WT macrophages (Fig. 2G). While macrophages typically secrete TNF-α in concert with other proinflammatory cytokines, we speculate that TNF-α could potentially be secreted through an unconventional pathway in TLR2-KO cells, explaining the different results for this cytokine. In support of this notion, microglia and tumor cells were described to release TNF-α through an alternative vesicular shedding pathway that does not require de novo gene transcription.^68–70^ We further showed that, unlike their WT counterparts, animals lacking TLR2 showed a similar sepsis phenotype following sleep interruption versus normal sleep, with comparable mortality and little difference in their disease score or serum cytokine levels (Fig. 3). Our data indicate that sleep interruption aggravates sepsis through an effect that is largely TLR2 dependent.

While TLR2 played a dominant role in this context, it should be noted that multiple pattern recognition receptors contribute to CLP sepsis. Both gram-positive and gram-negative bacteria are released from the cecum, and multiple damage-associated molecular patterns (DAMPs) are released from host cells, including HMGB-1^71^^,^^72^ and DNA.^73^ Interestingly, while TLR2-KO macrophages produced no IL-6 and MCP-1 in response to cecal bacteria in vitro, these cytokines were still produced in the sepsis model, likely in response to gram-negative bacteria and DAMPs.

Does sleep interruption induce long-term changes in animal physiology driving this phenotype, or are these effects quickly reversed? We found that the detrimental effects of sleep interruption were almost completely reversed following 48 hours of recovery sleep and fully reversed after 7 days (Fig. 4B). These results imply that sleep interruption is more likely to affect cytokine production and sepsis by inducing transient changes in gene expression, rather than epigenetic changes, which are typically long-lasting.

How does sleep interruption alter macrophage gene expression to influence their function? Although our data suggest that TLR2 is required for this effect, its expression levels were not altered by sleep interruption (Fig. S6), implying that the pathway is modulated through a different mechanism. To address this, we performed an unbiased transcriptome analysis comparing peritoneal macrophages of mice with interrupted sleep versus normal sleep and identified 680 differentially regulated genes (Fig. 5). The most highly ranked DEG was Car4, which was significantly upregulated in macrophages from animals subject to sleep interruption versus normal sleep (Fig. 5B). Notably, a prior report by Hudalla et al. identified a role of carbonic anhydrases in macrophage function.^74^ The authors showed that the carbonic anhydrase inhibitor ethoxzolamide suppressed both M1 and M2 activation and inhibited the production of IL-6 and TNF-α by cultured macrophages.^74^ Hence, the upregulation of Car4 after sleep interruption likely contributes to the increased cytokine production by macrophages, and more research should be performed in the future to test this.

Further analysis of our data revealed multiple pathways relating to cytokine–cytokine receptor signaling (Fig. 6), which may also contribute to the increased cytokine production that we observed (Figs. 1 and 2). As well, we uncovered other genes relating to host defense against bacteria, cell killing, and antimicrobial peptides that are altered by sleep interruption (Fig. 6). Therefore, it appears that multiple components of macrophage biology are affected by sleep, leading to additive and synergistic effects that are expected to contribute to the increased sepsis severity and higher mortality rates. Keller et al. previously showed that >8% of the macrophage transcriptome oscillates with circadian rhythms.^75^ Sleep could potentially affect these diurnal gene expression patterns, as well as directly alter gene expression independent of the circadian system. Notably, our macrophages were harvested from mice immediately after lights on (ZT-0; the same timing as when sepsis was induced in our earlier experiments). In the future, it would be interesting to examine how sleep affects gene expression throughout the 24-hour cycle by sampling additional timepoints throughout the day.

Taken together, our data suggest a model whereby sleep interruption alters the macrophage transcriptome, affecting the expression of multiple genes that regulate inflammation. This “immunological priming” results in a greater cytokine response to the subsequent CLP sepsis challenge, an effect that is mediated by TLR2. Excessive levels of cytokines seemed to drive the increased sepsis mortality after sleep interruption in our model. In tandem, sleep interruption increased the production of IL-10 and increased leukocyte apoptosis, exacerbating the detrimental immunosuppressive component of sepsis. Fortunately, however, 48-hour recovery sleep reversed the immunological priming effect, and improved animal survival. Our research has also revealed changes in gene expression that may explain how sleep interruption alters macrophage biology, including upregulation of Car4 and altered cytokine-receptor signaling. More research is needed to further elucidate these effects.

What were the strengths and weaknesses of our study? The strengths included the large sample size, inclusion of a control procedure to assess off-target effects, inclusion of both males and females, analysis of sex as a biological variable for the initial experiments, the inclusion of mechanistic approaches (TLR2-KO mice), testing for longevity of the effect (sleep recovery experiments), and the use of transcriptome analysis to broadly interrogate changes in macrophage gene expression in an unbiased fashion. An important limitation of our study was the fact that sleep was interrupted only before sepsis induction, and not afterward. This approach was selected to isolate the immune priming effects of sleep interruption. However, hospitalized patients may experience sleep interruption before, during, and after sepsis. Notably, the study by Friese et al. interrupted sleep only after CLP, and this also exacerbated sepsis.^39^ In the future, it will be important to perform more research looking at the effects of sleep interruption before, during, and after sepsis, to get a more complete picture of its ongoing effects. Other limitations of our study include the fact that our cohort did not include enough males and females to power a sex comparison for every experiment (due to time and funding constraints). Also, while blinding was used wherever practical, this was not possible in every experiment due to fluctuations in the study team over time. Finally, this study used a preclinical animal model. While mice remain a highly relevant model to study sepsis, some important differences do exist between this species and humans (eg mice are nocturnal while humans are diurnal, and people have more neutrophils in their bloodstream than mice). Future clinical studies should be performed to verify the clinical relevance of our findings.

In summary, our study has provided new fundamental evidence that sleep interruption, similar to that experienced by hospitalized patients, increases vulnerability to severe infections by rewiring the macrophage inflammatory response. More research is needed to elucidate further details of these effects. Ultimately, this investigation could reveal biomarkers of poor sleep to identify specific patients at greater risk, and flag them for follow-up to monitor and improve their sleep. Moreover, some of the genes or pathways affected by sleep interruption may be druggable, providing a potential opportunity to reverse their effects when a good night’s sleep remains beyond our grasp.

## Supplementary Material

vkag130_Supplementary_Data

## Data Availability

Raw RNA-seq reads (FASTQ), count matrices, and associated metadata have been deposited at the National Center for Biotechnology Information (NCBI) under BioProject accession PRJNA1439358, with Sequence Read Archive (SRA) accession numbers SRR37679236–SRR37679251.

## References

[1] Ramar K et al Sleep is essential to health: an American Academy of Sleep Medicine position statement. J Clin Sleep Med. 2021;17:2115–2119. 34170250 10.5664/jcsm.9476 PMC8494094

[2] Miyazaki S , Liu CY, Hayashi Y. Sleep in vertebrate and invertebrate animals, and insights into the function and evolution of sleep. Neurosci Res. 2017;118:3–12. 28501499 10.1016/j.neures.2017.04.017

[3] Rechtschaffen A , Gilliland MA, Bergmann BM, Winter JB. Physiological correlates of prolonged sleep deprivation in rats. Science. 1983;221:182–184. 6857280 10.1126/science.6857280

[4] Borbély AA , Daan S, Wirz-Justice A, Deboer T. The two-process model of sleep regulation: a reappraisal. J Sleep Res. 2016;25:131–143. 26762182 10.1111/jsr.12371

[5] Huang L et al Functions and mechanisms of adenosine and its receptors in sleep regulation. Sleep Med. 2024;115:210–217. 38373361 10.1016/j.sleep.2024.02.012

[6] Walker WE. Goodnight, sleep tight, don’t let the microbes bite: a review of sleep and its effects on sepsis and inflammation. Shock. 2022;58:189–195. 35959798 10.1097/SHK.0000000000001976 PMC9489678

[7] Singer M et al The third international consensus definitions for sepsis and septic shock (Sepsis-3). JAMA. 2016;315:801–810. 26903338 10.1001/jama.2016.0287 PMC4968574

[8] Heipertz EL et al Circadian rhythms influence the severity of sepsis in mice via a TLR2-dependent, leukocyte-intrinsic mechanism. J Immunol. 2018;201:193–201. 29760192 10.4049/jimmunol.1701677 PMC9351006

[9] Silver AC , Arjona A, Walker WE, Fikrig E. The circadian clock controls toll-like receptor 9-mediated innate and adaptive immunity. Immunity. 2012;36:251–261. 22342842 10.1016/j.immuni.2011.12.017 PMC3315694

[10] Sinton CM , Kovakkattu D, Friese RS. Validation of a novel method to interrupt sleep in the mouse. J Neurosci Methods. 2009;184:71–78. 19646474 10.1016/j.jneumeth.2009.07.026

[11] Garcia LF , Singh V, Mireles B, Dwivedi AK, Walker WE. Common variables that influence sepsis mortality in mice. J Inflamm Res. 2023;16:1121–1134. 36941984 10.2147/JIR.S400115 PMC10024505

[12] Walker WE. Methods to study the innate immune response to sepsis. Methods Mol Biol. 2018;1717:189–206. 29468593 10.1007/978-1-4939-7526-6_15

[13] Ali T et al Creatine kinase is elevated by the submandibular vein bleed technique, obscuring the measurement of muscle damage in sepsis. Shock. 2025;63:944–946. 40367917 10.1097/SHK.0000000000002585 PMC12105962

[14] Heipertz EL , Harper J, Walker WE. STING and TRIF contribute to mouse sepsis, depending on severity of the disease model. Shock. 2017;47:621–631. 27755506 10.1097/SHK.0000000000000771

[15] Walker WE , Bozzi AT, Goldstein DR. IRF3 contributes to sepsis pathogenesis in the mouse cecal ligation and puncture model. J Leukoc Biol. 2012;92:1261–1268. 23048204 10.1189/jlb.0312138 PMC3501894

[16] Goswami DG et al Large peritoneal macrophages and transitional premonocytes promote survival during abdominal sepsis. Immunohorizons. 2021;5:994–1007. 34965966 10.4049/immunohorizons.2100086

[17] Goswami DG , Walker WE. Detection of blood cell surface biomarkers in septic mice. Methods Mol Biol. 2021;2321:191–205. 34048018 10.1007/978-1-0716-1488-4_17

[18] Kim D , Langmead B, Salzberg SL. HISAT: a fast spliced aligner with low memory requirements. Nat Methods. 2015;12:357–360. 25751142 10.1038/nmeth.3317 PMC4655817

[19] Liao Y , Smyth GK, Shi W. featureCounts: an efficient general purpose program for assigning sequence reads to genomic features. Bioinformatics. 2014;30:923–930. 24227677 10.1093/bioinformatics/btt656

[20] Love MI , Huber W, Anders S. Moderated estimation of fold change and dispersion for RNA-seq data with DESeq2. Genome Biol. 2014;15:550. 25516281 10.1186/s13059-014-0550-8 PMC4302049

[21] Wu T et al clusterProfiler 4.0: a universal enrichment tool for interpreting omics data. Innovation (Camb). 2021;2:100141. 34557778 10.1016/j.xinn.2021.100141 PMC8454663

[22] Kelly LS , Darden DB, Fenner BP, Efron PA, Mohr AM. The hematopoietic stem/progenitor cell response to hemorrhage, injury, and sepsis: a review of pathophysiology. Shock. 2021;56:30–41. 10.1097/SHK.0000000000001699 PMC8141062 33234838

[23] Cohnheim J. Neue Untersuchungen über die Entzündung. A. Hirschwald; 1873.

[24] Hotchkiss RS Jr. , et al Sepsis-induced apoptosis causes progressive profound depletion of B and CD4+ T lymphocytes in humans. J Immunol. 2001;166:6952–6963. 11359857 10.4049/jimmunol.166.11.6952

[25] Hotchkiss RS et al Apoptotic cell death in patients with sepsis, shock, and multiple organ dysfunction. Crit Care Med. 1999;27:1230–1251. 10446814 10.1097/00003246-199907000-00002

[26] Chaudhry H et al Role of cytokines as a double-edged sword in sepsis. In Vivo. 2013;27:669–684. 24292568 PMC4378830

[27] Price AE et al A map of toll-like receptor expression in the intestinal epithelium reveals distinct spatial, cell type-specific, and temporal patterns. Immunity. 2018;49:560–575.e6. 30170812 10.1016/j.immuni.2018.07.016 PMC6152941

[28] Ates D , Tire Y, Mermer A, Kozanhan B. The effect of preoperative sleep quality on postoperative delirium in middle-aged and elderly patients: a clinical setting. J Coll Physicians Surg Pak. 2026;36:147–152. 41689312 10.29271/jcpsp.2026.02.147

[29] Sun T et al Sleep and circadian rhythm disturbances in intensive care unit (ICU)-acquired delirium: a case-control study. J Int Med Res. 2021;49:300060521990502. 33730927 10.1177/0300060521990502 PMC7983249

[30] Liu C et al Assessment of sleep quality using cardiopulmonary coupling and its predictive value for delirium in ICU patients. Sleep Med. 2025;126:222–227. 39705984 10.1016/j.sleep.2024.12.020

[31] Marchasson L et al Impact of sleep disturbances on outcomes in intensive care units. Crit Care. 2024;28:331. 39385194 10.1186/s13054-024-05118-4 PMC11466020

[32] Elías MN et al Nighttime sleep duration is associated with length of stay outcomes among older adult survivors of critical illness. Dimens Crit Care Nurs. 2020;39:145–154. 32251163 10.1097/DCC.0000000000000411 PMC11110929

[33] Knauert MP et al Association between death and loss of stage N2 sleep features among critically ill patients with delirium. J Crit Care. 2018;48:124–129. 30179762 10.1016/j.jcrc.2018.08.028 PMC6226351

[34] Willinger CM , Waddell KJ, Arora V, Patel MS, Ryan Greysen S. Patient-reported sleep and physical function during and after hospitalization. Sleep Health. 2024;10:249–254. 38151376 10.1016/j.sleh.2023.12.001 PMC11045314

[35] Krueger JM , Pappenheimer JR, Karnovsky ML. Sleep-promoting effects of muramyl peptides. Proc Natl Acad Sci U S A. 1982;79:6102–6106. 6964403 10.1073/pnas.79.19.6102 PMC347061

[36] Krueger JM , Walter J, Dinarello CA, Wolff SM, Chedid L. Sleep-promoting effects of endogenous pyrogen (interleukin-1). Am J Physiol. 1984;246:R994–R999. 6611091 10.1152/ajpregu.1984.246.6.R994

[37] Hart BL. Biological basis of the behavior of sick animals. Neurosci Biobehav Rev. 1988;12:123–137. 3050629 10.1016/s0149-7634(88)80004-6

[38] Angus DC et al Epidemiology of severe sepsis in the United States: analysis of incidence, outcome, and associated costs of care. Crit Care Med. 2001;29:1303–1310. 11445675 10.1097/00003246-200107000-00002

[39] Friese RS , Bruns B, Sinton CM. Sleep deprivation after septic insult increases mortality independent of age. J Trauma. 2009;66:50–54. 19131805 10.1097/TA.0b013e318190c3a1

[40] Gong S et al Dynamics and correlation of serum cortisol and corticosterone under different physiological or stressful conditions in mice. PLoS One. 2015;10:e0117503. 25699675 10.1371/journal.pone.0117503 PMC4336318

[41] Miyawaki T et al Circadian changes of T lymphocyte subsets in human peripheral blood. Clin Exp Immunol. 1984;55:618–622. 6608426 PMC1535929

[42] Born J , Lange T, Hansen K, Mölle M, Fehm HL. Effects of sleep and circadian rhythm on human circulating immune cells. J Immunol. 1997;158:4454–4464. 9127011

[43] He W et al Circadian expression of migratory factors establishes lineage-specific signatures that guide the homing of leukocyte subsets to tissues. Immunity. 2018;49:1175–1190.e7. 30527911 10.1016/j.immuni.2018.10.007 PMC6303219

[44] Lange T , Dimitrov S, Born J. Effects of sleep and circadian rhythm on the human immune system. Ann N Y Acad Sci. 2010;1193:48–59. 20398008 10.1111/j.1749-6632.2009.05300.x

[45] Dimitrov S , Lange T, Nohroudi K, Born J. Number and function of circulating human antigen presenting cells regulated by sleep. Sleep. 2007;30:401–411. 17520784 10.1093/sleep/30.4.401

[46] Le Tulzo Y et al Early circulating lymphocyte apoptosis in human septic shock is associated with poor outcome. Shock. 2002;18:487–494. 12462554 10.1097/00024382-200212000-00001

[47] Zhang Y et al PD-L1 blockade improves survival in experimental sepsis by inhibiting lymphocyte apoptosis and reversing monocyte dysfunction. Crit Care. 2010;14:R220. 21118528 10.1186/cc9354 PMC3220038

[48] Chang K et al Targeting the programmed cell death 1: programmed cell death ligand 1 pathway reverses T cell exhaustion in patients with sepsis. Crit Care. 2014;18:R3. 24387680 10.1186/cc13176 PMC4056005

[49] Vgontzas AN et al Circadian interleukin-6 secretion and quantity and depth of sleep. J Clin Endocrinol Metab. 1999;84:2603–2607. 10443646 10.1210/jcem.84.8.5894

[50] Redwine L , Hauger RL, Gillin JC, Irwin M. Effects of sleep and sleep deprivation on interleukin-6, growth hormone, cortisol, and melatonin levels in humans. J Clin Endocrinol Metab. 2000;85:3597–3603. 11061508 10.1210/jcem.85.10.6871

[51] Iwakura Y , Ishigame H. The IL-23/IL-17 axis in inflammation. J Clin Invest. 2006;116:1218–1222. 16670765 10.1172/JCI28508 PMC1451213

[52] Liu X et al Sleep loss potentiates Th17-cell pathogenicity and promotes autoimmune uveitis. Clin Transl Med. 2023;13:e1250. 37132178 10.1002/ctm2.1250 PMC10154899

[53] McDermott AJ et al Interleukin-23 (IL-23), independent of IL-17 and IL-22, drives neutrophil recruitment and innate inflammation during *Clostridium difficile* colitis in mice. Immunology. 2016;147:114–124. 26455347 10.1111/imm.12545 PMC4693884

[54] Chen Q et al Triggering receptor expressed on myeloid cells-2 protects aged mice against sepsis by mitigating the IL-23/IL-17A response. Shock. 2021;56:98–107. 32991795 10.1097/SHK.0000000000001668

[55] Tanaka T , Narazaki M, Kishimoto T. IL-6 in inflammation, immunity, and disease. Cold Spring Harb Perspect Biol. 2014;6:a016295. 25190079 10.1101/cshperspect.a016295 PMC4176007

[56] Riedemann NC et al Protective effects of IL-6 blockade in sepsis are linked to reduced C5a receptor expression. J Immunol. 2003;170:503–507. 12496437 10.4049/jimmunol.170.1.503

[57] Remick DG , Bolgos G, Copeland S, Siddiqui J. Role of interleukin-6 in mortality from and physiologic response to sepsis. Infect Immun. 2005;73:2751–2757. 15845478 10.1128/IAI.73.5.2751-2757.2005 PMC1087378

[58] Remick D , Siddiqui J, Osuchowski M, Nemzek J. Six at six, the inflammatory response to sepsis. Shock. 2006;25:12.16369180

[59] Naffaa M et al Interleukin-6 at discharge predicts all-cause mortality in patients with sepsis. Am J Emerg Med. 2013;31:1361–1364. 23896015 10.1016/j.ajem.2013.06.011

[60] van Loo G , Bertrand MJM. Death by TNF: a road to inflammation. Nat Rev Immunol. 2023;23:289–303. 36380021 10.1038/s41577-022-00792-3 PMC9665039

[61] Bianconi V , Sahebkar A, Atkin SL, Pirro M. The regulation and importance of monocyte chemoattractant protein-1. Curr Opin Hematol. 2018;25:44–51. 28914666 10.1097/MOH.0000000000000389

[62] L’Heureux M , Kashiouris M, Fowler A, Fisher B. Association between IL-10 and mortality in sepsis-induced ARDS. Chest. 2020;158:A621.

[63] Wu HP et al Serial cytokine levels in patients with severe sepsis. Inflamm Res. 2009;58:385–393. 19262987 10.1007/s00011-009-0003-0

[64] Rau M et al Clinical manifestations but not cytokine profiles differentiate adult-onset Still’s disease and sepsis. J Rheumatol. 2010;37:2369–2376. 20810496 10.3899/jrheum.100247

[65] Surbatovic M , Filipovic N, Radakovic S, Stankovic N, Slavkovic Z. Immune cytokine response in combat casualties: blast or explosive trauma with or without secondary sepsis. Mil Med. 2007;172:190–195. 17357775 10.7205/milmed.172.2.190

[66] Gogos CA , Drosou E, Bassaris HP, Skoutelis A. Pro-versus anti-inflammatory cytokine profile in patients with severe sepsis: a marker for prognosis and future therapeutic options. J Infect Dis. 2000;181:176–180. 10608764 10.1086/315214

[67] Latifi SQ , O’Riordan MA, Levine AD. Interleukin-10 controls the onset of irreversible septic shock. Infect Immun. 2002;70:4441–4446. 12117955 10.1128/IAI.70.8.4441-4446.2002 PMC128185

[68] Raffaele S , Lombardi M, Verderio C, Fumagalli M. TNF production and release from microglia via extracellular vesicles: impact on brain functions. Cells. 2020;9:2145. 32977412 10.3390/cells9102145 PMC7598215

[69] Xie B-W et al Tumor-derived extracellular vesicles delivering TNF-α promotes colorectal cancer metastasis via the NF-κB/LAMB3/AKT axis by targeting SNAP23. Arch Biochem Biophys. 2023;741:109605. 37086961 10.1016/j.abb.2023.109605

[70] Fitzgerald W et al A system of cytokines encapsulated in extracellular vesicles. Sci Rep. 2018;8:8973. 29895824 10.1038/s41598-018-27190-x PMC5997670

[71] Wang H et al HMG-1 as a late mediator of endotoxin lethality in mice. Science. 1999;285:248–251. 10398600 10.1126/science.285.5425.248

[72] Yang H et al Reversing established sepsis with antagonists of endogenous high-mobility group box 1. Proc Natl Acad Sci U S A. 2004;101:296–301. 14695889 10.1073/pnas.2434651100 PMC314179

[73] Dwivedi DJ , et al; Canadian Critical Care Translational Biology Group. Prognostic utility and characterization of cell-free DNA in patients with severe sepsis. Crit Care. 2012;16:R151. 22889177 10.1186/cc11466 PMC3580740

[74] Hudalla H et al Carbonic anhydrase inhibition ameliorates inflammation and experimental pulmonary hypertension. Am J Respir Cell Mol Biol. 2019;61:512–524. 30951642 10.1165/rcmb.2018-0232OC PMC6775956

[75] Keller M et al A circadian clock in macrophages controls inflammatory immune responses. Proc Natl Acad Sci U S A. 2009;106:21407–21412. 19955445 10.1073/pnas.0906361106 PMC2795539

