# Supplementary material for: Sleep interruption aggravates sepsis by rewiring the macrophage immune response
Source: J Immunol. 2026 Jun 8;215(6):vkag130. doi: 10.1093/jimmun/vkag130 (PMC13244266; doi:10.1093/jimmun/vkag130)
Supplement: vkag130_Supplementary_Data [file vkag130_supplementary_data.pdf]

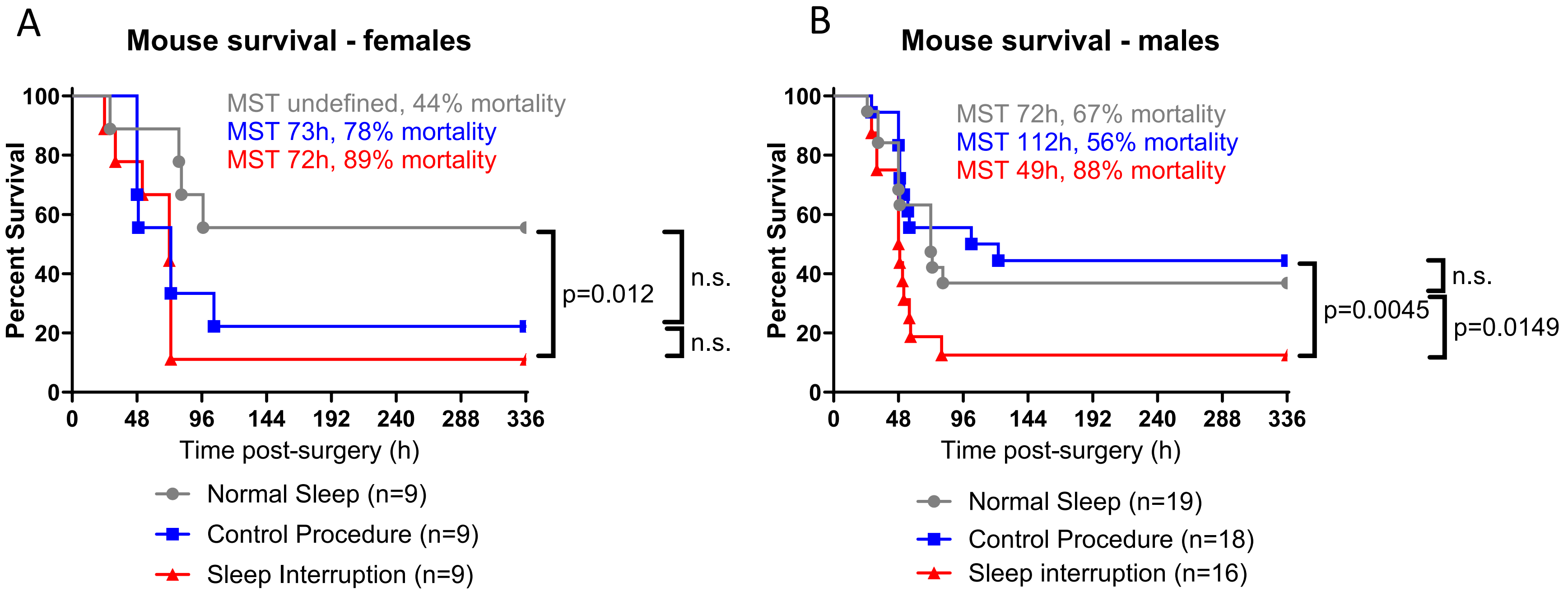

Figure S1. Sleep interruption exacerbates sepsis in males and females. Female and male C57BL/6 mice were subject to sleep interruption, a control procedure, or allowed normal sleep, and subsequently sepsis was induced by CLP, per the scheme shown in Figure 1A. These graphs show mouse survival by sex for (A) females and (B) males. n.s. = not significant.

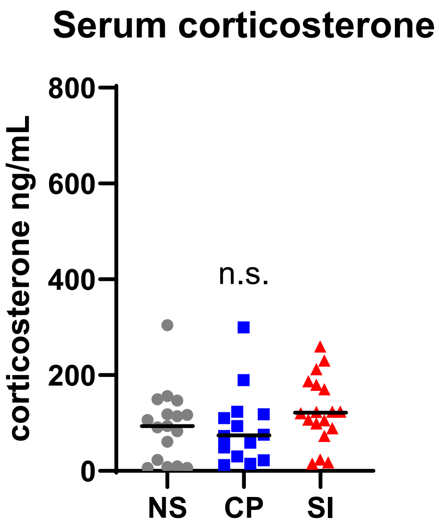

Figure S2. Corticosterone levels in mice. C57BL/6 mice were subject to sleep interruption (SI), a control procedure (CP), or allowed normal sleep (NS). Subsequently serum was obtained and the corticosterone levels were quantified. Graph shows individual values and the median. Data were compared with a Kruskall-Wallis test, and no significant differences were observed. n.s. = not significant.

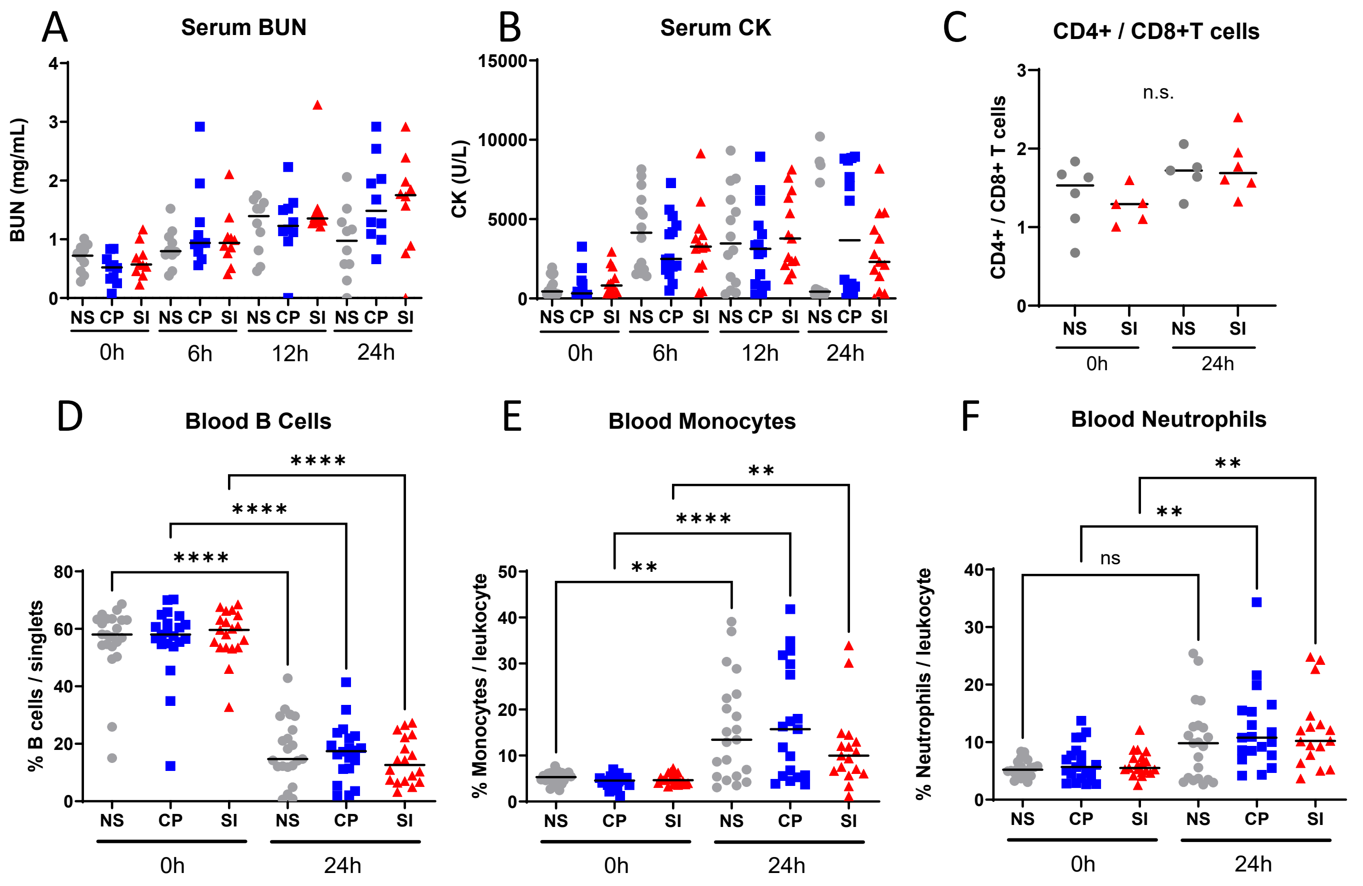

Figure S3. Examining the effects of sleep interruption on additional serum markers of organ dysfunction, and blood leukocyte populations. Female and male C57BL/6 mice were subject to sleep interruption (SI), a control procedure (CP), or allowed normal sleep (NS), and subsequently sepsis was induced by CLP, per the scheme shown in Figure 1A. Blood samples were collected at 0h (after sleep interruption, before CLP), and at 6, 12 and 24h post-CLP. At each time point, we measured markers of organ dysfunction, including (A) Blood urea nitrogen (BUN), a marker of kidney damage, and (B) creatine Kinase (CK), a marker of skeletal muscle damage. At 0h and 24h, we also stained the cells with antibodies and used FACS analysis to determine the percentage of (C) CD4/CD8 T cells, (D) B cells, (E) Ly6C+Ly6G- Monocytes, and (F) Ly6C+Ly6G+neutrophils. The levels of Blood B cells, monocytes and neutrophils were compared with a Kruskal Wallis test, followed by Duns multiple comparison test (we preformed comparisons between conditions at each time point, and the p values are shown; comparisons between the groups at each time point were not significant). n.s. = not significant.

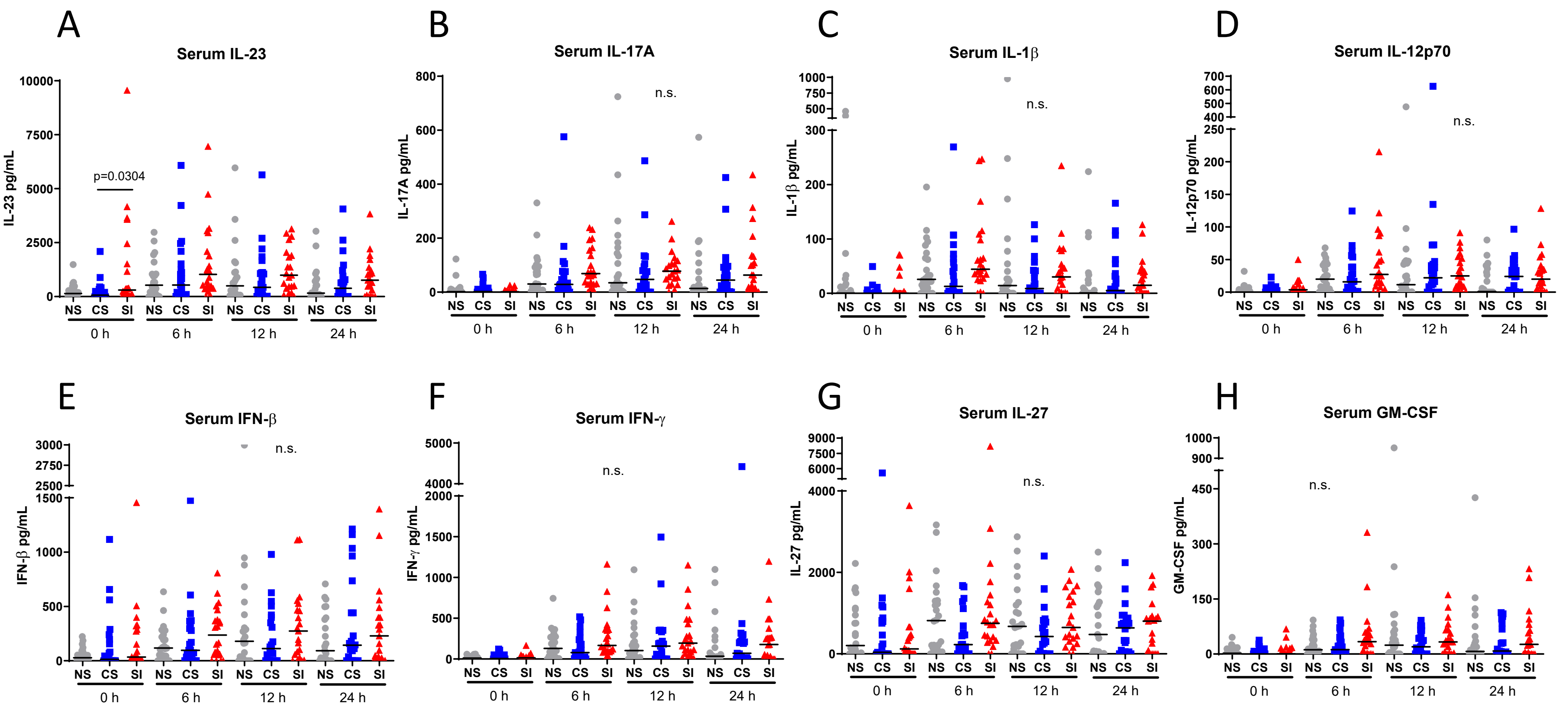

Figure S4. Examining the effects of sleep interruption on additional serum cytokines. Mixed sex C57BL/6 mice were subject to sleep interruption (SI), a control procedure (CP), or allowed normal sleep (NS), and subsequently sepsis was induced by CLP, per the scheme shown in Figure 1A. Blood samples were collected at 0h (after sleep interruption, before CLP), and at 6, 12 and 24h post-CLP. At each time point, serum cytokines were quantified, including (A) IL-23 (p=0.035 at 0h for the Kruskal-Wallis test), (B) IL-17A, (C) IL-1β, (D) IL-12p70, (E) IFN-β, (F) IFN-γ, (G) IL-27, and (H) GM-CSF. Data were compared with a Kruskal-Wallis test (p value is reported above for IL-23 at 0h, and the rest were not significant). A Dunn's multiple comparison test was also performed, and multiplicity adjusted p values are shown on the figure. n.s. = not significant.

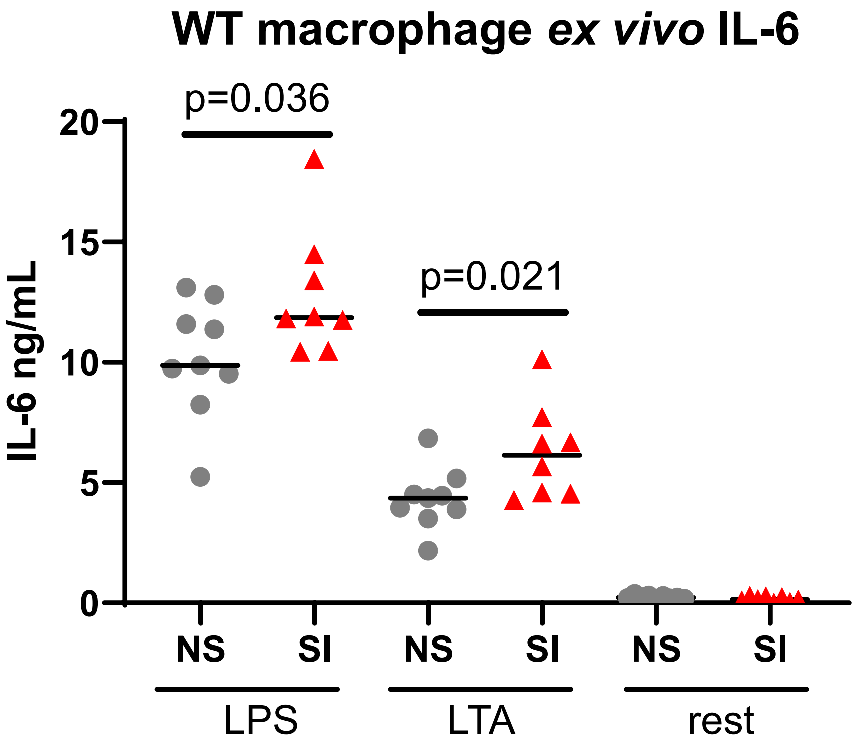

Figure S5: Sleep interruption increases macrophage production of cytokines in response to LPS and LTA. (A) Male and female C57BL/6 mice (n=6/group) were subject to sleep interruption (SI) or allowed normal sleep (NS). Peritoneal macrophages were harvested and cultured *ex vivo* with LPS, LTA, or media only (rest) for 4h. The media was harvested after 4h and we used ELISA to quantify IL-6. Each data point represents a single biological replicate. Data were compared with a Mann Whitney test for each agonist. n.s. = not significant.

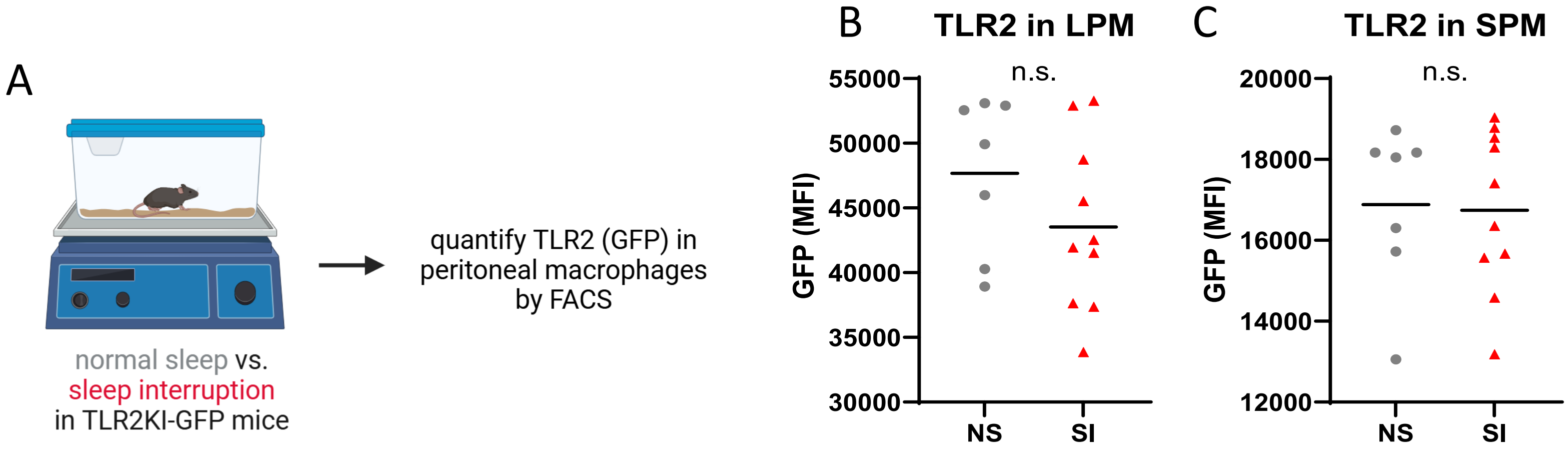

Figure S6. Examining the effects of sleep interruption on TLR2 expression levels. Female and male TLR2KI-GFPmice were subject to sleep interruption (SI), or allowed normal sleep (NS), and then peritoneal lavage was collected. Using antibody staining and FACS, we examined GFP as a marker of TLR2 expression within (A) F4/80-hi, MHCII-lo large peritoneal macrophages (LPM) and (B) F4/80-int/lo, MHCII-hi small peritoneal macrophages (SPM). Graphs show median fluorescence intensity. n.s. = not significant.

Table S1. DEGs relating to the defense response to bacterium (GO enrichment, padj=0.033)

Gene	Function/role	log2FoldChange	Fold change	P value
**Lipocalin-2 (Lcn2)**	Lcn2 sequesters iron to limit bacterial growth. It also exerts anti-inflammatory functions and protects the body from cell and tissue stress (Jaberi et al. Biomed Pharmacother 142:112002, 2021).	0.82	1.77	0.00
**triggering receptors expressed by myeloid cells 3 (Trem3)**	TREMs are activating receptors on macrophages, that associate with DAP-12. TREM1 and TREM2 are known to modulate TLR signaling, while the function or TREM3 is less clear (Chung et al. Eur J Immunol. 32: 59, 2002; Colonna et al. Nat Rev Immunol 23:580, 2023).	0.86	1.82	0.00
**Purinergic Receptor P2X7 (P2rx7)**	P2rx7 senses extracellular ATP released from damaged cells. Also, regulates ATP release from cytosolic stores (Johnsen at al., Pur Signal 15:155, 2019).	-0.43	0.74	0.00
**Interleukin-7 receptor (Il7r)**	IL7R regulates tissue-resident macrophage development (Leung et al. Development 146:dev176180, 2019). It plays a key role in the development, differentiation and survival of T cells and innate lymphoid cells (Barata et al. Nat Immunol 20:1584, 2019).	-0.53	0.69	0.01
**2',5'-Oligoadenylate Synthetase 1 (Oas1c)**	Oas1c is induced by interferons (IFNs) and degrades viral RNA (Choi et al. Exp Mol Med 47:e144, 2015).	-0.50	0.71	0.01
**peptidoglycan recognition protein 1 (Pglyrp1)**	Pglyrp1 helps the host detect peptidoglycan. It exerts bactericidal activity and promotes inflammation (Dziarski et al. Innate Immun 16:168, 2010).	0.52	1.43	0.02
**Lactotransferrin (Ltf)**	Ltf is an iron binding protein present in neutrophil granules and milk. It sequesters iron to inhibit bacterial growth and exerts anti-inflammatory activity. (Cao et al. Front Nutr 9:1018336, 2022).	1.23	2.34	0.02
**2610528A11Rik**	This is an orphan gene associated with skin inflammation (Dainichi et al. Front Immunol 13:825032, 2022).	1.88	3.68	0.02
**Whey Acidic Protein/Four-Disulfide Core Domain 21 (Wfdc21)**	Wfdc21 regulates the production of inflammatory factors in LPS-treated cells (Xie et al. Med Sci Monit 24:4054, 2018).	2.06	4.16	0.03
**Guanylate-Binding Protein 5 (Gbp5)**	Gbp5 is an interferon inducible factor that promotes inflammasome assembly (Shenoy et al. Science 336:481, 2012).	1.22	2.33	0.03
**Haptoglobin (Hp)**	Hp is an acute phase protein that binds to free hemoglobin and detoxifies it (di Masi et al. Mol Asp Med73: 100851, 2020)	0.56	1.47	0.03
**Guanylate-binding protein 2 (Gbp2)**	Gbp2 is an IFN-inducible GTPase that promotes inflammasome activation, helping to clear infected cells during the immune response (Kim et al. J Biol Chem 283:9157, 2012).	0.48	1.39	0.03
**Hematopoietic Cell Kinase (Hck)**	Hck is an Src-family tyrosine kinase that regulates cell proliferation and survival (Lantermans et al. Leukem 35:881, 2021).	0.34	1.27	0.03
**Proteoglycan 2 (Prg2)**	Prg2 controls neuron branch growth (Brosig et al. Cell Rep. 29:2028, 2019). It is upregulated in pregnancy and in systemic lupus erythematosus (Qiao et al. Clin Immunol 239:109042, 2022).	2.92	7.59	0.04
**H2-Q10**	H2-Q10 is a non-classical MHC protein that acts as a ligand for the inhibitory Ly49C receptor (Sullivan et al. J Biol Chem 291:18740, 2016).	1.24	2.36	0.04
**Cluster of differentiation 4 (CD4)**	CD4 is the co-receptor of the T cell receptor and plays a key role in CD4+ T cell activation. It also mediates macrophage differentiation, function and cellular interactions. Soluble CD4 prevents excessive TLR activation during sepsis (Zhang et al. Signal Trans Target Ther 8:236, 2023).	-0.52	0.70	0.04
**Galectin 4 (Lgals4)**	Lgals4 is an epithelial cell lectin that mediates wound healing and intestinal inflammation among other processes (Cao et al. Protein Cell. 7:314, 2016).	-0.44	0.74	0.04
**Iigp1**	Iligp1 is an interferon-inducible GTPase that disrupts the vacuolar membrane surrounding intracellular parasites (Uthaiah et al. J Biol Chem 278: 29336, 2003; Pawlowski et al. BMC Biol 9:7, 2011).	1.57	2.97	0.05
**cytochrome b-245 alpha chain (Cyba)**	Cyba is a subunit of NADPH oxidase, which produces reactive oxygen species for host defense (Panday et al. Cell and Mol Immunol 12:5, 2015).	0.22	1.16	0.05

Table S2. DEGs relating to cell killing (GO enrichment, padj=0.051)

Gene	Function/role	log2FoldChange	Fold change	P value
**Neurotensin (Nts)**	Nts increases stimulates production of nitric oxide via the JAK-STAT pathway in LPS-treated cells (Kim et al. Neuropeptides 40:221, 2006).	2.40	5.28	0.00
**Triggering receptors expressed by myeloid cells 3 (Trem3)**	TREMs are activating receptors on macrophages that associate with DAP-12. TREM1 and TREM2 are known to modulate TLR signaling, while the function or TREM3 is less clear (Chung et al. Eur J Immunol. 32: 59, 2002; Colonna et al. Nat Rev Immunol 23:580, 2023).	0.86	1.82	0.00
**Thymus- and activation-regulated chemokine (TARC/Ccl17)**	TARC modulates T cell development in the thymus as well as the trafficking and activation of T cells, particularly Th2 T cells and Tregs (Imai et al. J Biol Chem 271:21514, 1996). It is elevated in inflammatory and autoimmune diseases. (Wenzel et al. J Invest Dermatol 124:1241, 2005).	-0.87	0.55	0.00
**Purinergic Receptor P2X7 (P2rx7)**	P2rx7 senses extracellular ATP released from damaged cells and regulates ATP release from cytosolic stores (Johnsen at al. Purinergic Signal 15:155, 2019).	-0.43	0.74	0.00
**Interleukin-7 receptor (Il7r)**	IIL7R regulates tissue-resident macrophage development (Leung et al. Development 146:dev176180, 2019). It plays a key role in the development, differentiation and survival of T cells and innate lymphoid cells (Barata et al. Nat Immunol 20:1584, 2019).	-0.53	0.69	0.01
**killer cell lectin-like receptor subfamily B member 1C (Klrb1c)**	Klrb1c promotes NK and NKT cell cytotoxicity and stimulates INF-g production during immune response. (Rozbeský et al. Molecules.20:3463, 2015).	-0.69	0.62	0.01
**Deoxyribonuclease I (DNase1)**	DNase1 degrades extracellular DNA released by apoptotic cells to limit the immune response (Lauková et al. Biomolecules 10:1036, 2020).	-0.53	0.69	0.01
**chemokine ligand 8/ MCP-2 (Ccl8)**	CCL8 attracts monocytes, T cells, NK cells, basophils and eosinophils to the site of inflammation, and activates these cells (Gong et al. J Biol Chem 273:4289, 1998).	2.70	6.48	0.02
**peptidoglycan recognition protein 1 (Pglyrp1)**	Pglyrp1 helps the host detect peptidoglycan. It exerts bactericidal activity and promotes inflammation (Dziarski et al. Innate Immun 16:168, 2010).	0.52	1.43	0.02
**Lactotransferrin (Ltf)**	Ltf is an iron binding protein present in neutrophil granules and milk. It sequesters iron to inhibit bacterial growth and exerts anti-inflammatory activity. (Cao et al. Front Nutr 9:1018336, 2022).	1.23	2.34	0.02
**Guanylate-Binding Protein 5 (Gbp5)**	Gbp5 is an interferon inducible factor that promotes inflammasome assembly (Shenoy et al. Science 336:481, 2012).	1.22	2.33	0.03
**Cathepsin E (Ctsc)**	Ctsc is a protease found in the endosomal compartment of macrophages; involved in MHC-II antigen processing in B cells (Nishioku et al. J Biol Chem 7:4816, 2002).	0.43	1.35	0.03
**Guanylate binding protein 2 (Gbp2)**	Gbp2 is an interferon-inducible GTPase that promotes inflammasome activation, helping to clear infected cells during the immune response (Kim et al. J Biol Chem 283:9157, 2012).	0.48	1.39	0.03
**Histocompatibility 2, Q region locus 10 (H2-Q10)**	H2-Q10 is a ligand for NK cell receptor, Ly49C. It may play a role in the development of liver NK cells (Goodall et al. Immunol Cell Biol 97:326, 2018).	1.24	2.36	0.04
**hypoxanthine guanine phosphoribosyl transferase (Hprt)**	Hprt is involved in the purine salvage pathway; converts hypoxanthine and guanine into IMP and GMP (Torres et al. Orphanet J Rare Dis 2:48, 2007).	0.18	1.13	0.05
**Complement component 3 (C3)**	C3 is a core component of the complement system. Its cleavage products initiate and amplify complement activation. C3 fragments have a variety of effector functions such as chemotaxis activation, phagocytosis, adhesion and immune modulation. Accumulation of C3b triggers the membrane attack complex on the target cell (Ricklin et al. Immunol Rev 274:33, 2017).	0.54	1.46	0.05

Table S3. DEGs relating to Cysteine and Methionine Metabolism (KEGG enrichment, padj=0.70)

Gene	Function/role	log2FoldChange	Fold change	P value
**Gm7901**	Gm7901 is a pseudogene of 3-phosphoglycerate dehydrogenase (which makes serine).	-1.05	0.48	0.00
**Glycine N-methyltransferase (Gnmt)**	Gnmt plays a role in methionine breakdown, converting glycine to N-methylglycine and S-adenosylmethionine (AdoMet) to S-adenosylhomocysteine (AdoHcy). The Adomet to AdoHcy ratio also regulates DNA methylation, affecting gene expression. (Luka et al. J Biol Chem 284:22507, 2009).	0.89	1.85	0.01
**Glutamic-oxaloacetic transaminase 1 (Got1)**	Got1 plays a role in amino acid metabolism. It transfers an amino group between aspartate and glutamate. (Peng et al. Front Oncol 14, 2004)	-0.38	0.77	0.03
**Methionine synthetase (Mtr)**	Mtr converts homocysteine to methionine. (Watkins et al. Am J Hum Genet. 71:143, 2002).	-0.14	0.91	0.03
**Gm9347**	Gm9347 is a pseudogene of 3-phosphoglycerate dehydrogenase (which makes serine).	-0.73	0.60	0.04
**Serine Dehydratase Like (Sdsl)**	Sdsl catalyzes deamination of L-threonine and L-serine. (Ogawa et al. J Biol Chem 264:15818, 1989)	0.85	1.81	0.04
**Gm29050**	Gm29050 is a lncRNA gene.	-1.58	0.34	0.05

Table S4. DEGs relating to Cytokine-cytokine receptor interaction (KEGG enrichment, padj=0.70)

Gene	Function/role	log2FoldChange	Fold change	P value
**Growth differentiation factor 9 (Gdf9)**	Gdf89 is a member of the TGF-β family. It is known to regulates ovarian function and essential for normal folliculogenesis (Stocker et al. J Biol Chem 295:7981, 2020).	1.64	3.11	0.00
**Interleukin 36 gamma (Il36g)**	IL36γ is a member of the interleukin 1 superfamily. It protects mice from viral infections, by inducing the production of inflammatory cytokines and inhibiting macrophage apoptosis (Wein et al J Immunol 201:573, 2018; Gardner et al Cytokine 111:63, 2018). IL-36 influences the IL23/IL-17 axis (Traks et al. BMC Med genet 20:10, 2019) and was also shown to play a causative role in cancer-induced cachexia (Hayashi et al. Nat Commun 15:7662, 2024).	4.04	16.34	0.00
**thymus- and activation-regulated chemokine (TARC/Ccl17)**	TARC modulates T cell development in the thymus as well as the trafficking and activation of T cells, particularly Th2 T cells and Tregs (Imai et al. J Biol Chem 271:21514, 1996). It is elevated in inflammatory and autoimmune diseases. (Wenzel et al. J Invest Dermatol 124:1241, 2005).	-0.87	0.55	0.00
**Interleukin-27 (Il27)**	IL-27 inhibits IL-17 production and Th17 T cell differentiation. (Murugaiyan et al. J Immunol 183:2435, 2009).	1.35	2.55	0.00
**Interleukin-7 receptor (Il7r)**	IL7R regulates tissue-resident macrophage development (Leung et al. Development 146:dev176180, 2019). It plays a key role in the development, differentiation and survival of T cells and innate lymphoid cells (Barata et al. Nat Immunol 20:1584, 2019).	-0.53	0.69	0.01
**Fractalkine (Cx3cl1)**	CX3CL1 mediates leukocyte migration and infiltration (Rodrigeuz et al Int J Mol Sci 25:15, 2024).	-0.99	0.50	0.01
**monocyte chemotactic protein-2 (MCP-2/Ccl8)**	MCP-2 mediates immune cell recruitment and inflammation (Chavez et al. Cell Sig 134: 111951, 2025).	2.70	6.48	0.02
**Cxcr2**	CXCR2 is a receptor that recruits neutrophils to the site of inflammation. It also regulates recruitment of macrophages to injured nerves (Jiang et al. Mol Immunol 169: 50, 2024).	3.93	15.23	0.02
**Predicted gene 15819 (Gm15819)**	Gm15819 is a lncRNA.	-1.53	0.35	0.02
**Interleukin 2 receptor subunit gamma (Il2rg)**	IL2rγ is the common gamma chain subunit of multiple cytokine receptors including IL-2, IL-4, IL-7, IL-9, IL-15, and IL-21 (Lin et al. Cold Spring Harb Perspect Biol 10:a028449, 2018).	0.25	1.19	0.02
**Cluster of differentiation 4 (Cd4)**	CD4 is the co-receptor of the T cell receptor and plays a key role in CD4+ T cell activation. It also mediates macrophage differentiation, function and cellular interactions. Soluble CD4 prevents excessive TLR activation during sepsis (Zhang et al. Signal Trans Target Ther 8:236, 2023).	-0.52	0.70	0.04
**Bone Morphogenetic Protein 6 (Bmp6)**	Bmp6 mediates bone and cartilage formation. It changes macrophage morphology, inhibits their proliferation, and induces inducible nitric oxide synthase and TNF-a (Hong et al Immunology 128:e442, 2009). It also inhibits proliferation of B and T cells (Kersten et al. BMC Immunol 6:9, 2005, Silvertsen et al. Eur J Immunol 37:2937, 2007).	0.69	1.61	0.04
**Interleukin 31 Receptor A (Il31ra)**	IL-31RA is a subunit of the IL-31 receptor. IL-31 signaling induces inflammation and itching (Datsi et al. Allergy 76:2982, 2021).	2.02	4.04	0.05

Table S5. DEGs relating to antimicrobial peptides (Reactome pathway enrichment, p=0.0013)

Gene	Function/role	log2FoldChange	Fold change	P value
**Lipocalin-2 (Lcn2)**	Lcn2 sequesters iron to limit bacterial growth. It also exerts anti-inflammatory functions and protects the body from cell and tissue stress (Jaberi et al. Biomed Pharmacother 142:112002, 2021).	0.82	1.77	0.00
**MRP8 (S100a8)**	S100a8 is a subunit of calprotectin, a calcium binding protein that mediates antimicrobial activity by sequestering transition metals (Rosen et al. Trends in Microbiol 30:654, 2022). It modulates the inflammatory response by stimulating neutrophil infiltration and induces a cytokine response via TLR4 activation (Wang et al. Front in Immunol 9:1298, 2018).	2.05	4.14	0.00
**MRP14 (S100a9)**	S100a9 is another subunit of calprotectin, a calcium binding protein that mediates antimicrobial activity by sequestering transition metals (Rosen et al. Trends in Microbiol 30:654, 2022). It modulates the inflammatory response by stimulating neutrophil infiltration and induces a cytokine response via TLR4 activation (Wang et al. Front in Immunol 9:1298, 2018).	3.38	10.40	0.01
**peptidoglycan recognition protein 1 (Pglyrp1)**	Pglyrp1 helps the host detect peptidoglycan. It exerts bactericidal activity and promotes inflammation (Dziarski et al. Innate Immun 16:168, 2010).	0.52	1.43	0.02
**Lactotransferrin (Ltf)**	Ltf is an iron binding protein present in neutrophil granules and milk. It sequesters iron to inhibit bacterial growth and exerts anti-inflammatory activity. (Cao et al. Front Nutr 9:1018336, 2022).	1.23	2.34	0.02
**Copper transport protein antioxidant-1 (Atox1)**	Atox1 acts as a copper chaperone in the secretory pathway and as a copper-dependent transcription factor that mediates cell proliferation (Itoh et al. J Biol Chem 283:9157, 2008).	0.28	1.22	0.04
**Cluster of differentiation 4 (Cd4)**	CD4 is the co-receptor of the T cell receptor and plays a key role in CD4+ T cell activation. It also mediates macrophage differentiation, function and cellular interactions. Soluble CD4 prevents excessive TLR activation during sepsis (Zhang et al. Signal Trans Target Ther 8:236, 2023).	-0.52	0.70	0.04
